# Macrobiont: Cradle for the Origin of Life and Creation of a Biosphere

**DOI:** 10.3390/life10110278

**Published:** 2020-11-12

**Authors:** Benton C. Clark, Vera M. Kolb

**Affiliations:** 1 Space Science Institute, Boulder, CO 80301, USA; 2 Department of Chemistry, University of Wisconsin—Parkside, Kenosha, WI 53141, USA; kolb@uwp.edu

**Keywords:** macrobiont, pond, prebiotic chemical evolution, origin of life, systems analysis, foreshore, mudflat, mud cracks, organics, transition elements, CHNOPS, hydrothermal

## Abstract

Although the cellular microorganism is the fundamental unit of biology, the origin of life (OoL) itself is unlikely to have occurred in a microscale environment. The macrobiont (MB) is the macro-scale setting where life originated. Guided by the methodologies of Systems Analysis, we focus on subaerial ponds of scale 3 to 300 m diameter. Within such ponds, there can be substantial heterogeneity, on the vertical, horizontal, and temporal scales, which enable multi-pot prebiotic chemical evolution. Pond size-sensitivities for several figures of merit are mathematically formulated, leading to the expectation that the optimum pond size for the OoL is intermediate, but biased toward smaller sizes. Sensitivities include relative access to nutrients, energy sources, and catalysts, as sourced from geological, atmospheric, hydrospheric, and astronomical contributors. Foreshores, especially with mudcracks, are identified as a favorable component for the success of the macrobiont. To bridge the gap between inanimate matter and a planetary-scale biosphere, five stages of evolution within the macrobiont are hypothesized: prebiotic chemistry → molecular replicator → protocell → macrobiont cell → colonizer cell. Comparison of ponds with other macrobionts, including hydrothermal and meteorite settings, allows a conclusion that more than one possible macrobiont locale could enable an OoL.

## 1. Introduction

The concept of the Macrobiont (MB) [1,2,3] is the planetary setting in which the first spark of life was struck from inanimate matter. Before this, each appropriate candidate setting was simply a potential macrobiont (pMB). Once biological activity was triggered, the transition to ever-more-complex and eventually sophisticated life forms could occur by the process of Darwinian evolution. This same setting is also the cradle for the development of a primitive, so-called protocell [4,5,6,7,8], and its prospective evolution. 

Investigations into the chemical origin of life face all the complexities of systems chemistry [9,10,11]. However, much recent progress has been made in laboratory demonstrations of relatively straight-forward pathways for prebiotic chemical evolution, with good yields of fundamental biomolecules to transition from plausible, simpler geological and atmospheric constituents. This progress has spanned various research groups, variously associated with, e.g., Bada, Benner, Carell, Deamer, Joyce, Sutherland, Szostak, Wächtershäuser [12,13,14,15,16,17,18,19], and many others. These have included studies of properties of aqueous media which are plausible according to our current understanding and inferences for environmental conditions on the early Earth. The properties generally include the availability of essential organic and inorganic feedstocks and in some cases, the availability of solar UV and other energy sources. There is also an expectation that favorable dynamic changes in the environments in which these reactions could occur all fall within the realm of credibility given our understandings of planetary environments.

Because any biological entity, ranging from the most fundamental cell to multicellular organisms, is clearly a combination of various critical components, the principles of Systems Analysis [20,21,22,23] can be applied. Application of this discipline could also provide insights into candidate macrobionts. The recent report of the U.S. National Academy of Sciences on “An Astrobiology Strategy for the Search for Life in the Universe” (2019) advocates the greater use of systems analysis in multiple areas of the field of Astrobiology [24]. As they state, “Astrobiology seeks to understand the web of interrelationships and feedbacks between time-variable planetary processes—both physical and chemical—and the proto-biological, chemical and organizational dynamics that led to the emergence and persistence of life. Systems science provides a holistic, transdisciplinary paradigm for addressing this complexity.”

The purpose of systems analysis is a formal inquiry into the nature of any complex entity that is best analyzed as a system made up of multiple entities, all of which interact between themselves and are essential to achieve its overall function. The developing macrobiont can be considered one such system.

A variety of possible starting environments have been suggested for where and how the origin of life (OoL) occurred, with some nebulous but others embracing more specific and constrained concepts. Not all settings are equally suitable to become a pMB. Its characteristics need to include access to H_2_O, to certain fundamental chemicals (organic compounds, including heteroatomic molecules or their precursors; CHNOPS elements plus Mg, K; certain transition metal elements) and to one or more energy sources (redox, photo, thermal) that can be transformed into useful chemical energy. Although we will address a variety of such settings, our primary focus in this analysis will be ponds, with emphasis on favorable geophysical characteristics (size, shape, connectivity), geochemical environments (access to feedstocks), and a range of activity levels (from quiescent to highly dynamic).

## 2. Methods and Approach

The first task in the systems approach is to define the primary function of the system and what are its requirements. We chose to primarily focus these analyses on the pond concept for our macrobiont. We define the pond as any small body of liquid water in a gravitational trap at the surface of a planetary object, providing interfaces with both atmospheric and geologic matter. 

For the body of water to be at least metastable, the planetary object must be a planet or satellite that is sufficiently large and warm that it gravitationally enables an atmospheric pressure adequate to prevent liquid water from rapidly dissipating by boiling or escaping into space. An envelope of atmospheric gases also has the advantage of potentially providing some of the needed nutrients and energy sources to the macrobiont.

In a typical systems analysis, the “Requirements” of the system are defined at the highest level possible, and then various sub-requirements are developed in a tree-branch scheme to subsequent lower levels. Our top-level requirement was for the macrobiont to provide the spatial and dynamic environments of the macrobiont that facilitate the origin of life and further development of cellular organisms for creation of a global biosphere.

It was Darwin himself, of course, who famously mused that some “warm little pond” may have been where life got its start [25].

The various definitions of “pond”, as opposed to “lake”, range from 20 ha to 80 ha (2–8 × 10^4^ m^2^) in surface area. In this analysis, we generalized by studying circular bodies of water within the range of 3 m to 300 m diameter (equivalent to 7 m^2^ to 7 × 10^4^ m^2^ subaerial area). The lower limit is in the “puddle” class, which are quantities so small as to likely be too ephemeral, although there are special environmental conditions which can be envisioned to prolong this longevity. A body of water only a few meters across may seem far too small to host the origin of living entities, but it is useful to consider that for a micron-sized microbe it would be equivalent to the relative size of the North Atlantic Ocean when compared to the size of a human being. As we have shown in Section 3, however, there are both advantages and disadvantages of pond size with respect to the promotion of the origin and propagation of life, such that the most optimum size is likely somewhere between these two limits for pond size.

### 2.1. Systems Analysis

The characterization of an assemblage of matter and energy can be studied from many different viewpoints. At some point, however, it becomes apparent that there may be properties of the assemblage that are more than the sum of its parts. Such an assemblage is called a “system.” It is now recognized that systems are more than just a human invention (e.g., a transportation system). Rather, there are interactions in nature that can best be described overall at a higher level than just of its isolated portions, e.g., a hydrologic system comprised of: ocean → clouds → rainfall → rivers → ocean.

There are a variety of perspectives that can be used to view any system. As seen in Table 1, these generalized perspectives [20] can be applied to the macrobiont and be used as a guide to the analyses that should be considered.

#### 2.1.1. System Versus Heap

Life as we know it (LAWKI) is based on organic molecules. The prebiotic chemical environment on early Earth may have been seeded by the components of carbonaceous asteroid meteorites and impacting comets [26,27,28,29]. It may have also been provided by atmospherically created organics [30,31]. In both cases, the organic milieu would have been a complex mixture of relevant compounds within an abundance of non-relevant molecules. Even if the molecules of life predominated, they would not have been organized into a functioning unit recognizable as life. From a systems analysis standpoint, such a non-functional conglomeration is known as a “heap” [21].

There is a critical difference between a heap and a system. A heap, in the astrobiological context, would be all the individual chemicals that are needed to create an organism, such as amino acids, proteins, carbohydrates, nucleic acids, membrane components, and so forth. Let us suppose that we had all of these on hand, and just mixed them together. This would create a heap of biochemical components. If we would take out some of them, such as some amino acids and some sugars, the heap would still be a heap, although slightly different.

Now, let us assume that we are applying this to a living organism, which contains all the compounds that were originally in the heap, but now the system is alive. What is the difference? The answer is that an alive organism is a system, in which all of the components interact among themselves in specific and complex ways, to create metabolic cycles, feedback loops, information networks, and so forth.

Such interactions may be called “communications”. In a system, if one severs such communications, for example breaks up a metabolic cycle, destroys the feedback loop pathways, or disables information networks, the system could stop functioning as such. Depending on the extent of damage in the communications between the parts in the system, the organism may not be alive anymore. Thus, a system does not remain a system if communications between its subsystems are no longer functioning, while a heap remains a heap, even if somewhat changed. Parts of a heap do not communicate among themselves to form networks and other complex interactions.

We can describe the types of complex interactions between an organisms’ parts and could define life based on these. Such a definition would be suitable and satisfactory for biology, but not for astrobiology. For the latter we must explain how interactions formed to transform a prebiotic heap into a biotic system. It should be noted that the idea that communication is a critical feature at all levels of life has been proposed by Witzany [32].

A foundational procedure of systems analysis is to determine the functional requirements for performance by the system and then lay out the essential fundamental blocks that will be needed. Even highly complex systems can be subjected to this analytical procedure, as long as the fundamental blocks can be identified. At the highest level, these blocks are termed subsystems, because they themselves often have their own complexities that involve smaller systems and components.

In analysis of the macrobiont as a system, we take advantage of comparison of a typical engineering system with the prokaryotic microbe as a system, and the macrobiont itself as a dynamic system. For our comparison engineering system, we choose a spacecraft: the planetary rover. We choose a rover because of its importance to Astrobiology on many fronts, coupled with the fact that its system requires the full panoply of the STEM disciplines (Science, Technology, Engineering, Mathematics) which are all also essential for the study of the origin of our biosphere. Not least of all reasons for choosing a rover is that the resources invested into space missions dwarfs that allocated for all other subfields of astrobiology, and of course, like the category of life colloquially known as “dinosaurs,” space projects also have strong educational as well as inspirational components (e.g., the Arts).

#### 2.1.2. Functional Block Diagrams

Layout of a Block Diagram provides a roadmap of subsystems with their interconnections and interrelationships. It also enables analysis of the characteristics of the subsystems and their components independent of other subsystems until an overall analysis is made of the total system. Any system, from a spacecraft all the way down to an individual microbial cell, can be modelled in terms of a block diagram.

**Figure 1:**
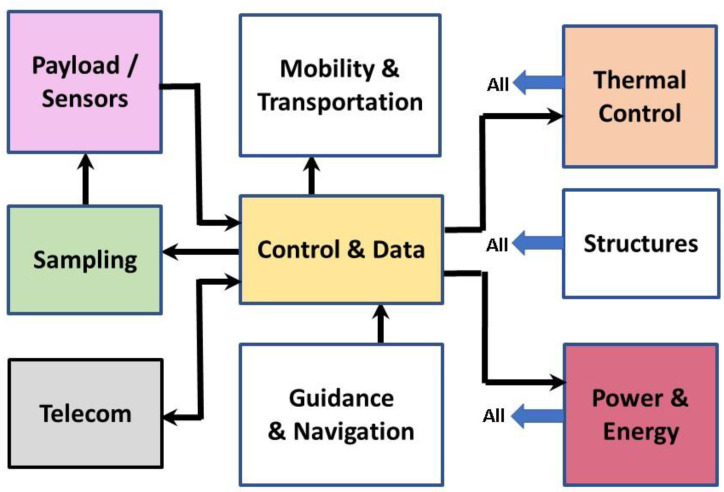
System block diagram (generic).

In Figure 1, a typical block diagram is shown for a spacecraft, in this case, applicable to our rover designed to explore the surface of a planet. 

There are 9 separate subsystems identified in this block diagram, but notable is that several are combinations of two generic functions. This occurs often because the specialists responsible for developing the subsystems are practitioners with expertise in both functions, because of their strong relationships to one another. Some of the functions have well-worn, proven implementations. For example, a computer which is sufficiently advanced for this rover application and is already “flight proven” because the same version was flown on a previous mission to the surface of Mars could be selected because of its “strong heritage” of application, even though recent developments may have revealed a computer which is faster and consumes less power. An analogy with biology could be, for example, a chemotactic response via motility that might be identical across multiple generations, until sufficient mutational changes occurred which could add sophistication to the stimulus detection apparatus and/or the responses.

If expanded, the 9 subsystems could be represented as 13 distinct subsystems. Not all subsystems of the rover system should be expected to have exact analogs in a primitive microbe’s system. After all, a planetary rover’s requirements and detailed design implementations benefit from the application of human intellect and technological developments. However, perhaps surprisingly, nearly all the same general functions are employed for life as for a spacecraft.

Both systems require a source of energy. For spacecraft, it is typically solar power, although nuclear thermoelectric energy is sometimes used instead, especially in deep space far from the sun or when roving on a dusty planet, such as Mars. Within the “Power subsystem” are the solar arrays or plutonium-238 energy sources, plus a battery and the necessary circuitry for distributing power to other subsystems and provide some conditioning of its parameters.

The beauty of the electrically powered rover, automobile, and smartphone is that it renders them agnostic of the source of energy that creates the intermediate, electricity, whether it is derived from wind, solar, or nuclear energy, or the energy released by combustion of petroleum or coal. In the biological world, the beauty of the diversities of metabolism is that different life forms can specialize in utilizing different sources of energy: from sunlight, or from the breakdown of organic compounds, or from one or more of a large variety of geochemical redox couples available in the natural environment. These metabolic energies are converted into ATP and NADH molecules, which serve as the “batteries” for life. These molecules are available to be utilized to power metabolism, as guided by the actions of enzymes rather than command over wires or telecommunication links.

The Control and Data handling functions in a spacecraft, including a rover, are best handled by a general-purpose computer with necessary circuitry to allow it to communicate with other subsystems. It provides the information flow outward for controlling the operation of the other subsystems and also an inward flow of data gathered by these subsystems. In many subsystems, there are microcomputers that interpret the control information and implement the instructions in more specific detail. Analogously, the central DNA library of instructions, combined with actions of regulatory RNA and proteins, orchestrate the activities of the biological organism. Like the rover, it also undergoes error detection and correction (EDAC) activities (via DNA replication and ribosomal proofreading steps) to assure that instructions are being implemented correctly.

Unlike a rover, however, the microbe is manufacturing copies of all its ingredients, in preparation for reproducing itself. True that the rover may obtain samples from the regolith and ingest it into its “mouth(s)”, by drilling or scooping. However, this is for purposes of analyzing the material and in some cases for encapsulating certain samples for eventual return to Earth for much more detailed analysis. The microbe is acquiring material for chemically processing it into appropriate molecules for manufacturing new membrane and cytoplasm components, until it has doubled its size, in preparation for fission into two near-identical organisms. This “Sampling” function is the acquisition of nutrients, mainly those atmospheric and geochemical constituents which are dissolved in the aqueous medium in which it thrives.

In spite of the fact that a rover has legs and feet (wheels), at least one arm, one ear, many eyes, a nose, and a mouth with a particularly aggressive proboscis (drill), it is not a form of life! Furthermore, although it is homeothermic and clearly exhibits complex behavior, it cannot reproduce itself, so it is not alive.

The rover traverses the terrain thanks to its wheels and suspension system. The microbe may use flagella to reorient and propel itself, or pili to achieve twitching motility, or fimbriae to anchor itself against an enveloping flow field.

A rover can use inertial measurement systems and wheel odometry, as well as image analysis to navigate its surroundings. A microbe can use its sensors and motility system for chemotaxis to detect gradients and guide movements to navigate toward nutrient sources, or phototaxis to navigate toward or away from a source of light.

Thermal control on a spacecraft is often a major issue and requires strong passive and often additional active controls to maintain temperatures within the operating and survival temperature ranges of each of its components. At the size of a microbe, there is no possibility of maintaining significant temperature differentials, but with an uncanny thermotactic response even to very small thermal gradients across the organism, it can swim in the direction of warmer or cooler temperatures. Moreover, microbes have optimum growth ranges, as well as larger temperature ranges for survival. In dormant forms, such as the endospore, they can endure cryogenic temperatures and can also survive desiccation.

A rover can communicate with Earth or a local orbiter with its radiofrequency transmitter and antennae. All spacecraft require telecommunication, both uplink and downlink, to receive instructions for future operations and to funnel back the critical compilations of data acquired by its cameras, sensors, and other scientific and housekeeping measurements.

The primary purpose of a rover mission is to gain unique new knowledge about the object being explored. Human ingenuity invented the wheel, vaccines, microscopes, and telescopes, but also the written language. The latter allows the distribution of this and other knowledge, as well as wisdom, to all humans, now and in the future (although it is not clear that these are always taken advantage of).

Some communities of organisms can use chemical-based excretions for signaling to enable the “quorum sensing” response triggered by high population densities. At the sub-cellular level, the genome must communicate its design and control information to the metabolic machinery, and every organism on Earth sends a one-way communication to each of its progeny, i.e., the genome copy that describes what set of genes has worked in the past (except for some changes, typically small, to try out). For rapid interactions, the microbe can take advantage of the speed of diffusion on the microscale.

Development of a new spacecraft design is based on the functional requirements plus design drawings created by the engineers. Originally called “blueprints” but now known as CAD (computer aided design) drawings, these are analogous to the genome of the cell. What we call “organs” in a multi-cellular organism, or organelles in the eukaryotic cell, are analogous to the “black boxes” in a spacecraft design. 

A rover needs structure for integrating all the “black boxes” that make up its subsystems; a microbe has its cytoskeleton and membranes. The rover needs mechanisms, including the motors to drive its wheels, move its arm, pan its science camera, and position its antenna. Structure is very important in all biological cells. Relevant to early life, apart from the structural intricacies of the later eukaryotic version of cellular life forms, there is a cytoskeleton that helps form overall shape, as well as a cell wall in many cases, and there are ribosomes. The latter are individual organelles but are composed not only of the rRNA molecule but also a group of well-coordinated proteins which do not apparently provide the primary enzymatic functionality but do provide a structural framework, which may enhance the efficiency of the rRNA. The ribosome is a mechanism that adds amino acids one-by-one as it advances the new polypeptide chain.

#### 2.1.3. Functional Block Diagram for the Generalized Macrobiont

Ironically, a living organism, the resilient cellular microbe, which is the ultimate product of the macrobiont, is rather more directly analogous to a rover, spacecraft, or other engineering system than is the macrobiont itself. For example, although there are energy flows in a pond MB, and modest storehouses of gravitational and thermal energy, these are not coordinated by any central controller, and although there is “Sampling”, it is nutrient acquisition by passive solution equilibration with atmospheric gases and alteration of local minerals already in contact with the pond water.

The MB has no propensity for self-mobility and therefore no need for guidance or navigation functions. However, within the pond MB, there is ample mobilization and transportation, driven by various natural forces, including thermally induced convection, winds, rainfall (including hail), etc. 

The MB does not communicate with other MBs (i.e., no “Telecom”), although we shall address also the possibility of interconnected ponds, each of which function partially as an independent type of MB.

Although ponds can have multiple thermal regimes, they reflect the strict physics of thermal energy flow and are not under any proactive control.

On the other hand, as will be discussed in detail in the next section, the MB is expected to have multiple functional components, separated physically and/or temporally, which combine to enable abiogenesis as well as an adequate set of internal environments for the development of capable cellular life forms. Whereas planetary rovers evolve more by changes in size and modernization than functional changes, and a free-living microbe has already invented a plethora of advanced biological capabilities, the MB must change with time to proceed in a large number of steps along the way from a heap of chemicals to the resultant sophisticated, compact, efficient, yet versatile living entities. These changes in the MB are accomplished by parallel evolutions, the one biological (Darwinian) and the other natural (environmental).

There are no active sensors from which decisions are made. In the spirit that the *raison d’être* of an exploratory planetary rover is its payload of instruments, it is the living organisms that arise in the MB which can be considered to constitute the “Payload” in the block diagram, and especially the cells which leave the MB and seed the flourishment of life in the outside world. Unlike the rover, but just as with the microbe, the macrobiont must manufacture its own “payload” itself.

### 2.2. Systems Analysis Methodologies

A hallmark of System Analysis is that it is, in principle, independent of scale and independent of discipline or field. Any given specific implementation is highly dependent on scale and technical details, but the analytical approach of defining performance characteristics of the System, and then selecting components, functions, and their interactions to meet those characteristics, can be applied at virtually any level of organization, from nano-scale molecular assemblages, to rockets, or to the giga-scale of planetary engineering.

Another hallmark is that there are a set of methodologies that are also independent of details. Studies aim to understand not only what the system’s performance is or would be in a wide range of environmental conditions; the system can be studied for performance as a function of incremental changes in various subsystems, to see how close it is to an ideal optimization; the system can be mathematically modeled to predict behaviors parametrically and evaluate the non-linearity of various responses; its resilience under stresses can be evaluated to assess its ability to return or remain within stable conditions in spite of changes in the environment around it (homeostasis); whether the system has one or more “tipping points” where its status and performance is forever changed by outside influences, and remains changed after those influences are relaxed (e.g., death of the organism); and evaluate lifetime against consumables (propellant for spacecraft; nutrients for organisms).

All of these analyses and appropriate tests are generally performed for expensive projects, such as developing a new spacecraft. Some cannot yet be performed for the macrobiont because the steps critical to full abiotic chemical evolution have not all been discovered, much less parametrically studied and reported. The latter are quite important. For example, how sensitive are various steps to salt concentration? Brines are a likelihood on most planets and depending on their composition and saturation level may be a hindrance to one or more critical syntheses (on the other hand, certain ions such as Mg^2+^ may provide an enabling environment). We apply sensitivity analyses to certain pond physical parameters and size-frequency distributions in what follows.

Such subsystems are conceived as quasi-self-contained, yet it is the task of systems engineering to assure that all functions and desired performance of the overall system are achieved. This is implemented by defining top-level Requirements on the overall System, and then determining from that what the Derived Requirements are on each of the subsystems. This hierarchy penetrates into detailed requirements of each subsystem and continues typically down to the 4th or 5th level of detail. For a typical project, this can result in ~10^3^ individual detailed requirements. As the design proceeds, there are processes of Verification and Validation (V&V) which demonstrate that each and every requirement is being met, typically by performing a test but in special cases by conducting appropriate analyses.

Certain mantra’s and guidelines are adopted during development of a system such as a spacecraft for planetary exploration. For example, the mantra “Better is the Enemy of Good Enough” expresses the objective of designing and implementing each subsystem such that it performs according to its derived requirements, but not significantly beyond (unless there is zero penalty for doing so). This guideline seeks to minimize cost and schedule risks by avoiding unnecessarily ambitious objectives or excessively elaborate solutions. This system imperative may not seem appropriate to the development of life forms, but the reality is that many organisms evolve by streamlining capabilities they may have had in the past in order to become more efficient at exploiting the specific environment they are in. As a macrobiont changes because of external influences, it may progress from its original role of providing the abode for rudimentary life to one for hosting cellular life, and this may be facilitated if certain aspects of its internal environment change in a favorable way. This will be examined in Section 4.

An engineering system, such as a Mars rover, will be stress-tested against various predicted environmental extremes (for example, the wide range of coldest winter night and warmest summer day for Mars, taking into account terrain vagaries, dust storms, etc.) For abiotic chemical evolution in the macrobiont, pathways which make demonstratable progress in the synthesis of key organic products and intermediates can be studied as a function of temperature, pH, Eh, catalytic ions, toxic or interfering molecules, and so forth.

In systems engineering design, specific functions are assigned to specific subsystems, and each subsystem may have more than one function. However, these assignments are not always universal or unique. In modern systems architectures for spacecraft, the central computer system generally does not directly operate the functions inside any given subsystem, but rather communicates operating information (“commands”) to a smaller, generally less capable microcomputer hosted inside the subsystem which then performs the details for the functions specified. Likewise, power is managed and maintained centrally, but the actual set of operating voltages within each subsystem are usually generated locally because different devices have different requirements for the actual voltage levels and their current-supply capabilities, as well as how accurate and stable those voltages need to be.

Thus, the subsystems are only semi-isolated, and there are often major interactions that must be controlled, such as in data generation and handling, in thermal control (one instrument’s warmth is another instruments noise-source), etc. It is also imperative that one subsystem’s operation not interfere or conflict with another subsystem. This necessitates special controls to prevent coupling of interference by electromagnetic waves emitted from operating circuits. In metabolic subsystems, it is important that key products or side products do not interfere with other metabolic pathways. 

#### Contaminants, Toxins, and Waste Products

Control of the environments in which components are built and a spacecraft is assembled can be critically important. This invokes the use of “clean rooms” and various cleaning procedures in manufacturing areas. Particle contamination of valves or sensitive surfaces can result in degraded performance or loss of mission. Organic contamination can interfere with mass spectrometry measurements and degrade optical sensors, especially for UV spectroscopy. Dust on lens surfaces can degrade IR spectrometers. Thermal absorption of solar insolation and re-radiation of heat can be seriously compromised by surface contamination, causing overheating. Organic and biological contamination can cause false positives in biosignature- or life-detection experiments.

Likewise, for a microbe to operate at peak efficiency, it must control its internal environment to maintain electrolyte balance, acquisition of nutrients, control of potentially toxic substances (including trace elements), and disposal of waste products. The MB must avoid or segregate side products, or “tars”, the so-called “asphalt problem” [13]. A 50% yield of a desired product in an abiotic synthesis is excellent, but not necessarily always enough. How to dispose of the rest? 

A planetary rover may not seem to create physical waste. Rocket propellant is used up and converted into waste gases, although these are ejected at the highest practical velocity in order to achieve maximum engine thrust. Covers and deployment arms are often released by one-shot pyrotechnic devices. Various other systems, such as rocket stages, the launch shroud, cruise stage, aeroshell, and parachute are all jettisoned at the appropriate stages during deployment to the surface of the target planet. Failure to dispose properly of any of these items will result in mission failure.

For the macrobiont, as well as the cell itself, the problem of wastes must be accommodated. Metabolic cycles provide a means to re-use critical intermediate molecules. Various degradation enzymes must be invented to catabolize unwanted or unneeded molecules into smaller units that are useful building block monomers that can be recycled, or versions that are easily discarded. In the MB itself, various forms of purification, including phase separations such as precipitation, flotation/flocculation, or crystallization can come into play.

### 2.3. Evolution of Systems

Systems can evolve in their design and implementation.

The automobile, motorcycle, and bicycle are various machines that convert other forms of energy into the mechanical energy of rotating wheels for movement of their center of mass. Shortly after the practical application of the internal combustion engine, the implementation of drive power for automobiles replaced their steam engines and electric motors. The spread of automobiles entered a phase of exponential growth and the replacement of other modes of transportation (e.g., horses) was rapid, while the growth of travel by rail greatly slowed. Although the automobile of today, especially the all-electric and computer infested versions, is radically different in implementation, its overall systems architecture is much the same, albeit with computer intelligence on a trend to replace the human intelligence that was originally an essential component.

In the earlier implementations, to start an automobile, a person had to “crank” the engine, and to start an airplane, one had to manually crank the propeller. Electric starters and all manner of other improvements to the various subsystems have made automobiles and airplanes the convenient, efficient, and even livable machines they are today.

Nevertheless, automobiles still utilize treaded wheels and a steering column to control where they travel. The lesson is that some subsystems can change radically while others may need only incremental improvements for the overall system to have greater functionality and become more robust. If a ribozyme was the first form of molecular replicator, it is an example of relatively straightforward and non-radical changes from the RNA World to the present biosphere’s extreme reliance on multiple versions of RNA molecules.

Evolution can be highly non-linear with time. The timeline of critical inventions for transportation technologies has had a remarkable regularity on a decadal time scale. From around 1860 when Étienne Lenoir developed the first commercially successful internal combustion engine, as well as a less successful automobile, until the automobile of Benz, took about two decades, and from then to the Wright-brothers first flight was another two decades. The first liquid-fueled rocket, by Robert Goddard, was successfully launched about another two decades later. One decade after that, the first jet engine-powered airplane came into being, and two decades later the first spacecraft was launched into orbit around Earth. From 1957, it was just a little over one decade until astronauts walked on the moon, and less than another decade later when the first Viking lander touched down on Mars. In just a bit over one century, our civilization transitioned from the rudimentary beginnings of horseless travel all the way to space travel, including to the surface of another planet.

The lesson from these achievements is that the stepwise and enormous, exponential gains in technological sophistication does not require exponential increases in time. Rather, logarithmically based measures of accomplishments in technologies can scale linearly with time increments. There are two obvious factors involved here: (1) the synergies and cumulative achievements of humankind have a multiplicative factor for increases in future accomplishments, and (2) the rapidly expanding tech-savvy workforce with time, result in a blooming complement of inventors and practitioners, all working in parallel with one another.

The chronological history of the biosphere since the origin of life appears to be similar, with an explosion of metabolic diversification versus geologic time, once life began. We believe this has occurred for the same two fundamental reasons, (1) as primitive cells developed a wider range of enzymes, more functional membranes, more efficient ribosomes, etc., they not only preserved all the accomplishments of past generations through propagation of the genome that recapitulated past successes, but they also (2) fueled the population explosion that resulted in wide distribution of talented new species and strains that now populate virtually the entire planet and explore simultaneously and independently the future mutation space and fitness landscape with greater speed than ever before. The rapid spread of antibiotic resistance is but one example.

A key component of the progress in human technologies has been the sharing of results, particularly those which enable success in achieving desirable capabilities. With early life, this is facilitated by horizontal gene transfer, as opposed to the selfish control of proprietary genes by higher species (equivalent to interminable patent control and trade secrets in human societies). As will be discussed in Section 4.2, it is just as likely that the stages of maturation and success of the macrobiont would have proceeded post-haste in evolutionary progress toward establishment of the planet-wide biosphere.

#### Complexity

A planetary rover is a complex system, but the living organism, even as embodied in a relatively simple bacterial or archaeal cell, is an even more complex system. Some of the characteristics of complex systems are the following: feedback loops; interdependent variables; metastable states; and non-Gaussian distributions of outputs. When not properly accounted for, each of these characteristics can individually produce system behavior that is not easily predictable and does not always follow simple deductive or statistical expectations. In the case of a system goal to enable the origin of living entities, complexities such as these can be advantageous because they provide a pathway to the desired goal, albeit relatively improbable. Darwinian evolution does not have a goal, per se, but the very creation of a subsystem that is actually capable of Darwinian evolution with natural selection may hinge on such complexities.

Technology systems such as a smartphone are extremely complex. Although a smartphone has more or less the same types of subsystems and same overall block diagram as the rover (except, relying on humans for transportation), it has the additional requirements that it must be extremely small, lightweight, and power efficient. As if the individual components were not already complicated enough, there is the problem that if a component part of one subsystem changes its design, it may affect not only that subsystem but others that use that same generic part (e.g., transistors, diodes, and integrated circuits).

In systems design, especially in modern approaches, this interplay of subsystems that leads to greater complexity is avoided where possible by minimizing the interconnectedness and interdependence of one subsystem on another, except where it is embedded in the primary functions. Darwinian evolution achieves this only rarely, and there is often an exquisite re-purposing of designs for multiple uses. In this sense, many highly evolved biological subsystems are inherently very complex and sometimes lead to absurd outcomes, in both implementations and behaviors. In the origin of life, there may have been stages that correspond well to what has been achieved in the laboratory by test-tube chemistry, but others which do not. Nevertheless, by the process of survival of the fittest, the latter were either discarded, replaced, or simply sufficiently refined such that they were suitable for the successful progression toward cellular life.

### 2.4. Incomplete Knowledge

When designing a complex system, especially for the first time or for a new environment, it is important to avoid the mistake of assuming that all decisions can be made without further investigation. Rather, to avoid costly delays or incorrect decision-making, the subsystems and the environments can be surveyed to identify where there is incomplete knowledge. These so-called “Knowledge Gaps” can then be circumscribed as to their possible extremes, to the extent possible, to determine how they might affect the implementation of the system. They can also become the target of intensive, new investigations in order to reduce the uncertainties in these extremes. For our rover, this can include regolith characteristics, such as soil bearing strength, energy dissipation, slope distributions, etc., and probabilistic occurrences of rocks of various sizes and angularity (including sharpness). For our macrobiont, this can include susceptibility of ponds to meteorological disturbances and influences on their longevity. For the origin of life itself, the main knowledge gaps are various pathways for prebiotic chemical evolution, such that the abiotic-to-biotic transition can occur.

Application of these techniques can be challenging, but also revealing. In Section 4, we shall examine ways in which systems analysis pertains to the MB.

## 3. Pond as Macrobiont

Conditions that can support habitability by known forms of life seems reasonable for consideration for where life might have originated in primordial times. Presumably, it was this same line of reasoning that led Darwin to suppose [25] that the lush conditions which support a flourishing biota in warm little ponds would also be an ideal locale for where life itself first emerged. Darwin was apparently not aware, it turned out, of the discovery of microbes by van Leeuwenhoek nearly two centuries earlier [33] nor of the contemporary breakthroughs in gene studies by Mendel nor the isolation of a molecule from a cell nucleus that would later become known as DNA, by Miescher.

**Figure 2:**
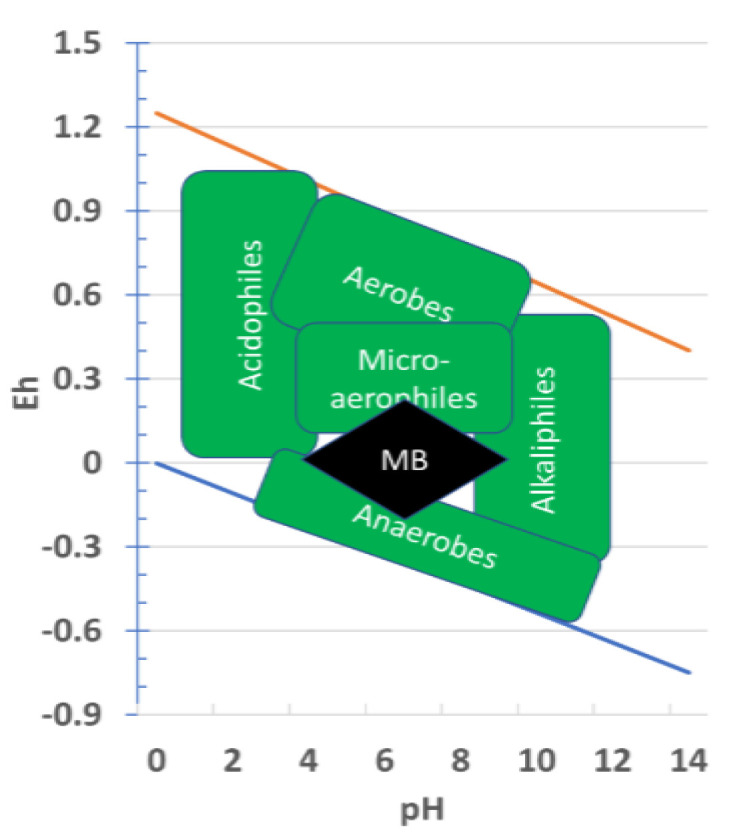
Pourbaix diagram for diverse life and the macrobiont.

Habitability of an environment is not an adequate criterion, however, to render it appropriate for the origin of the first life forms. Microorganisms have an enormous range of habitability, including those environments that actually are mostly uninhabitable but are nevertheless home to various extremophiles. In the end, microbes seem to be able to conquer virtually any environment that has a supply of liquid water and nutrients and at least one version of a whole array of usable energy sources. For example, as seen in the Pourbaix diagram of Figure 2, there are classes of organisms which can exploit virtually any regime in pH/Eh space. Nevertheless, the wide range of laboratory investigations into abiotic chemical evolution are generally favored by neutral conditions and moderate but occasional access to more extremes of pH. Although there is much to be done to explore how wide can be the limits under which abiotic chemical evolution proceeds for various syntheses, it is so far unreasonable to expect the zone to be much larger than indicated in this figure. This is one of the sensitivity analyses that could benefit from further study and also be better constrained by models of the potential ranges of Eh/pH in candidate MB environments on early Earth. 

Life on Earth is biochemically based, although not just ordinary chemistry but a variety of other factors, such as redox chemistry, control of catalysts, and multiple physical processes, all come into play. What was the environment that enabled the progression of events from abiotic chemical evolution to creating of the first encoded replicating system that enabled Darwinian evolution at the molecular level to proceed? In the laboratory, scientists use graduated test tubes, flasks, heat sources, stirring bars, and other apparatus to carefully control the various steps of abiotic chemical evolution being investigated. The macrobiont can be neither so intentional-minded nor precise. Nevertheless, it must somehow provide the means by which certain reactions can be favored in location or in time, only to be combined in a subsequent step with other ingredients or the products of other reactions. The laboratory studies can explore how various series of reactions can be melded together by changing as few parameters as possible so as to lessen the demands on the potential macrobiont’s repertoire.

A few choice environments would begin, at best, as potential macrobionts (pMB), until the dynamics of the pMB resulted in the creating of the first appropriate replicating unit. The macrobiont itself is not “alive” because it cannot reproduce itself. However, it is nonetheless the environment in which the replicating entity can arise and have the potential to prosper. To survive in and populate the external world, the replicator needs to evolve much further, presumably to eventually achieve the capabilities we normally associate with the free-living cell, the highly competent and widely regarded “fundamental unit of biology.”

In this section, we focus on the range of factors for a pMB, which form a system suitable for the creation of that first replicator and its supportive constructs. These factors include everything from the concentrations and species of molecules and ions, to the variety of dynamic forces and events, from whirlwinds to lightning.

Because it provides the widest range and most dynamic of possible environmental factors, we first consider a subaerial locale, the pond. In Section 5, we will invoke a hydrothermal component to the subaerial pond and also compare a suboceanic locale containing hydrothermal vents as well as hydrothermal crater lakes.

Because it lies at the intersection of atmospheric, geologic, and hydrologic forces, as well as contributions from astronomical objects (sun, meteorites), the pond environment is multi-faceted and also subject to the time history of its location. Examples of the extraordinarily wide range of potentially contributing forces and materials for a pond macrobiont is provided in Table 2.

### 3.1. Sizes and Populations of Ponds

Although system analysis can be mostly or even totally scale-independent, the actual embodiment of many subsystems does depend on features of scale. Life begins at the microscale (molecules), but it is highly likely that the macrobiont is a phenomenon of the macroscale. What scale-size is most germane to the MB? It has long been argued that an ocean is a difficult case, because its overwhelming size invokes dilutions and dispersions that severely dampen the desired chemical reactions. Concocting life from a mere puddle seems just as unlikely for other reasons, such as limited longevity and stifling uniformity. There is no precise definition of the sizes of “ponds” as compared to the range of puddle → pond → lake → sea, but smaller entities typically predominate.

Across many phenomena, the smaller entities are generally much more numerous. There are far more stars than galaxies, and many more planets than stars. Moons, asteroids and comets are far more numerous than planets. On a hybrid planet (land and sea), there are far more islands than continents. From the standpoint of a biosphere, there are more protists than metazoans; more prokaryotes than eucaryotes; many more ribosomes than cells; more RNA molecules than ribosomes; more small molecules than proteins; more electrons than molecules.

Likewise, we might expect that hydrologically, there are more lakes than oceans and more ponds than lakes. Whether there are more puddles than ponds in any given area will depend on the terrain but especially upon the persistence of wet weather compared to dry weather.

Gravity is a leveling force with respect to planet-scale terrain, whether achieved by low viscosity lavas or the erosive powers of flowing water, aeolian abrasion, or mass wasting. With bidirectionally undulating topography, as water levels rise due to springs or rainfall, the smallest reservoirs will be filled first until they breach the local topographic highs to merge and produce larger ponds or endorheic lakes.

In a world-encompassing study of the sizes of mapped bodies of water on Earth, the data were best fit by a power law for the occurrences of lakes of various areas [34]. We have converted these areas to a model diameter, to derive a power law for the integral size frequency distribution (ISFD) of circular pond diameters to

(1) N(>D)=kDβ

where D = diameter of the lake and N(>D) is the number of lakes with diameter greater than D.

**Figure 3:**
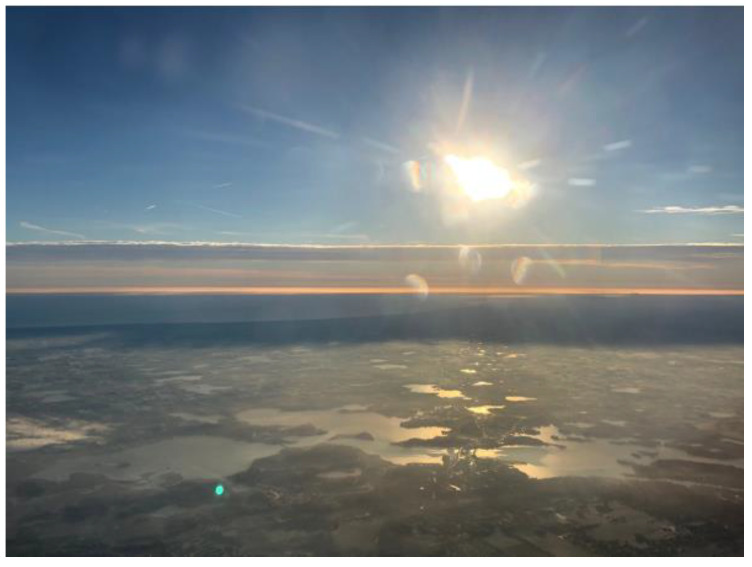
Airborne view of ponds and lakes populations, as revealed by favorable “sun-lake-observer” geometry. Photo credit: B. C. Clark.

Our fitted parameters are k = 5.91 × 10^11^ and β = −2.121. This is only slightly steeper than the ISFD which would correspond to equal areas of all lakes in equal size increments (β = −2.0). With this value of k, it is estimated that there are over 300 million bodies of water with D > 35 m on planet Earth. For lack of more data, we also use the same relationship down to smaller diameters (D = 3 m). Integrating to obtain the total area of these ponds up to D = 300 m, we find that 2.1% of dry land is under pond water. This may be an overestimate, and regional populations of ponds can vary by factors of up to four orders of magnitude [34], depending on the climate and hydrological state of the locale. However, as shown in Figure 3, with a more optimum vantage point than just from ground level, the density of water bodies can be observed to be quite high in many locations.

### 3.2. Shapes of Ponds

**Figure 4:**
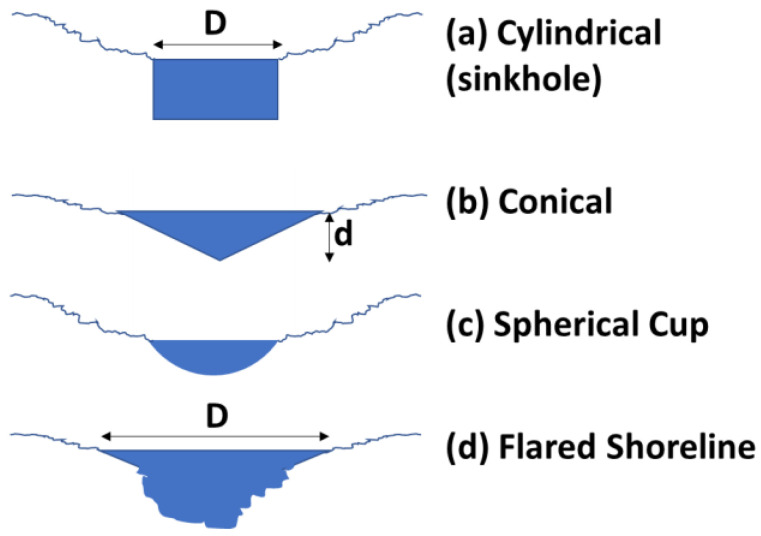
Example pond configurations (d = max depth, D = subaerial diameter, α = d/D).

The shape of the bottom surface of any specific pond can vary greatly. Although the original topography dictates the extent of the top planar surface, the geophysical mechanisms will dictate the shape of the surface in contact with pond water. Depressions in terrain can be tectonically related, or caused by many other processes such as uneven sedimentation, eolian or aqueous erosion, glacial carving, faulting, sediment sinkholes, impact cratering, caldera formation, etc. In the bottom surface, there can also be significant modifications with time because of sedimentation and erosive activities. For modeling purposes, we choose a variety of idealized bottom shapes, Figure 4. The inverted conical surface postulates a constant slope, whereas the spherical cup emulates the watch glass concavity for a fixed but offset radius of curvature. These well-known geometrical shapes allow calculations for shallow ponds with low-slope shorelines. In contrast, a cylinder model allows an approximation of a body of water with scarps for shorelines, such as in sinkholes from underground erosion, or calderas, or impact craters.

In all shapes, several key values and ratios can be readily calculated, and compared to one another to provide a range of possible variation. However, the most important and least constrained parameter is the depth-diameter relationship, which we capture as the inverse aspect ratio,

(2) α=d/D

where D = diameter of exposed surface, d = maximum depth of the pond.

Most values and ratios which parametrize the pond and its ingredients can be described as a function of α and D, as seen in Table 3. For additions of water to the pond, by rainfall, runoff, or groundwater, the increase in height is Δd. This table summarizes various pond attributes and how they scale, with the relevant formulae derived from the classical relationships for the selected geometric models.

### 3.3. Spatial Heterogeneity

In this report, we accept the conjecture that the origin of a colonizer-class cell could not arise from a single, homogeneous pot of primordial soup. Analogously, one would not expect that filling a heated cauldron with ore minerals and water and then bubbling through atmospheric gases for a semi-infinite amount of time could ever result in a planetary rover crawling out of the pot.

To realize a sufficient approximation to the multiple pot needs for sequential development of biologically critical ingredients, a pond can have subregions of differing environments and activities which are semi-sequestered for various periods of time.

Both horizontal and vertical sequestration can occur. These produce gradients in concentrations that allow separate, quasi-independent chemical evolution to occur.

The epitome of strong gradients is the rack of test tubes in the investigator’s lab, where each test tube has a different composition or concentration profile. This has its advantages for experimental studies because it can select reactions to favor desired outcomes by use of knowledge and insights into mechanisms and pathways. However, this approach is counter to that in the natural setting, where other reactions occur that are not useful to prebiotic chemistry.

Strong gradients are valuable in the multi-pot problem because it causes the reaction “pots” to be sufficiently isolated to favor different reactions and products. In addition to steep gradients in molecules and ions which favor certain reactions, there can be gradients in protons, electrons, and photons, resulting in redox or photochemically driven reactions that would not occur at all, or at least at the same rate, without them.

#### 3.3.1. Vertical Stratification

**Figure 5:**
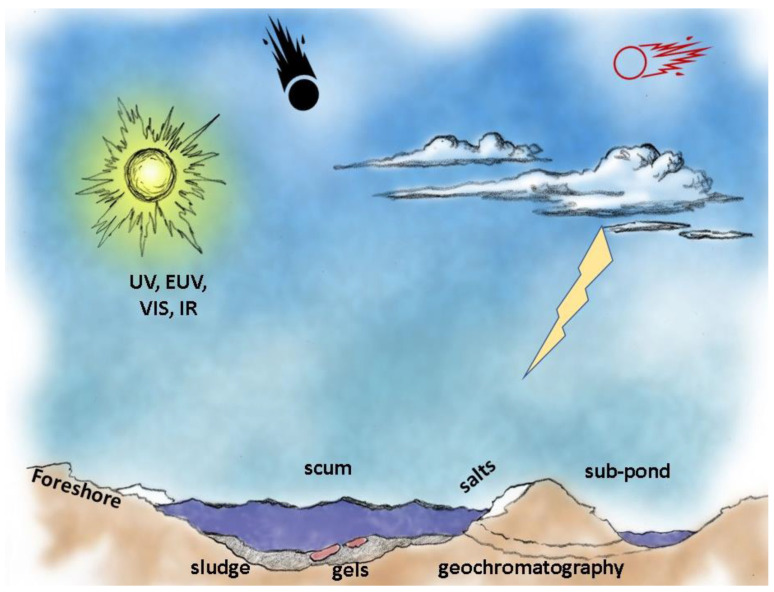
Vertical heterogeneity in a pond macrobiont. (painting credit: Michael Carroll).

Organic compounds and minerals which are soluble or in colloidal form will be more evenly dispersed in the pond water. However, the natural gravity gradient will result in the sorting out of those materials which are buoyant with respect to the density of pond water or its surface tension, forming a surface scum, as compared to those which sink to form a bottom sludge, Figure 5. Most rocks, soil, and dust grains which find their way into the pond will sink because mineral grains generally have densities far higher than H_2_O (e.g., 2 to 3+ g/cm^3^). Fine volcanic ash and pumice can be exceptions to sinking because of their high void volume. Dust depositions can be important sources of key nutrients in seawater [35] and lakes. 

Insoluble organic compounds which are of density greater than water will sink. In addition, less dense organic molecules may sink also if bound to denser material. This includes, for example, the insoluble organic matter (IOM) in meteorites that is attached to minerals.

This vertical stratification can be accentuated by ponds which are also thermally stratified (depths greater than several meters), such that the warmer epilimnion zone above their thermocline has a different circulation pattern than the colder hypolimnetic layer below. There is of course also solar insolation that is attenuated by the pond water. In a clear pond, sunlight can readily reach the bottom, but if the pond is turbid from colloidal matter, the euphotic zone can be sub-meter in depth. Penetration of UV radiation that might be needed for energetically enabled reactions, including the photochemical generation of oxidants [36] but can also be strongly affected by the nature of the pond material. In pure H_2_O, UV may reach meter depths, but the presence of small concentrations of salts, of certain organics, including the SOM (soluble organic matter) from Murchison meteorite, and/or Fe^2+^, the UV can be strongly attenuated in depths of just mm [37].

Not only day/night progression from diurnal cycles but also dark environments that may be dynamic can be created by photon attenuation from surface scum, from turbidity for material below the Secchi depth, or at locations in subsurface soil or beneath the bottom sludge. The forces of convection or Brownian motion can transport material up or down within the murky silt of a muddy pond.

#### 3.3.2. Horizontal Heterogeneity

**Figure 6:**
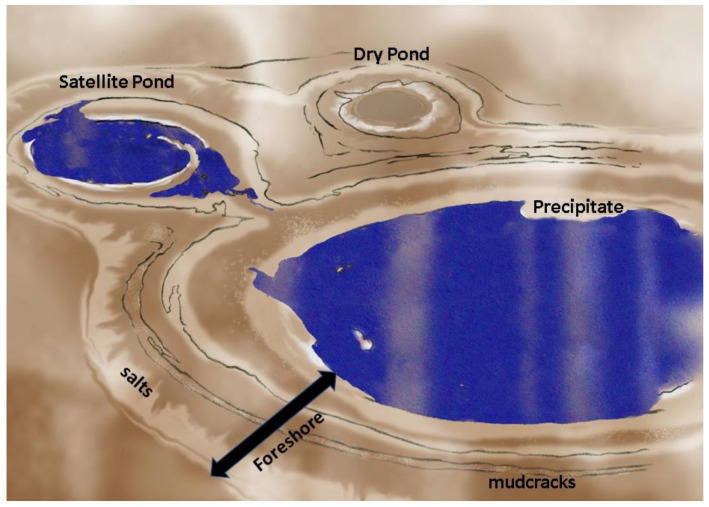
Horizontal heterogeneity in a pond macrobiont.

In addition to vertical stratification, there can be significant horizontal heterogeneity, capable of creating multiple semi-isolated regions at shorelines, Figure 6. One scenario is a very localized environment defined by the ruggedness of the shoreline. These invaginations can result in shorelines 2× or 3× as long as the circumference of a circular pond of equivalent area.

Bottom topography can also produce special regions of concentration. Prevailing winds can form preferred locations for concentrations, especially for surface foams and scums.

Because the rate of spread of the contents of these special regions by the process of diffusion alone is slow and progresses only as the inverse square of distance, it can operate as a semi-isolated component. If the pond medium is of high viscosity, due to high salts and/or organic contents, this semi-isolation can be even more effective.

Diffusion coefficients for large molecules, such as proteins or nucleic acid polymers are small (~100 µm^2^/s for 20 kDa tRNA or 30 kDa protein molecules, in water [38]). As derived from Fick’s law, the modeled times for these components to cross a 3 m pond are more than one century, and correspondingly longer by a factor of 10^4^ to traverse the diameter of our largest pond. However, even in apparently still water, these estimates are generally unrealistic because of subtle currents. Nonetheless, a significant concentration of a potential reactant A at one end a large quiescent pond, and another reactant B at the other end will experience low or no concentration of A and will react much more opportunistically with a reactant C that is nearby to it. Not only is there a lag time in translational diffusion, but the concentration will be dropping by the cube of the distances as the semi-isolated components proceed to diffuse closer to one another. Within the prokaryotic cell, because of their tiny size, and in spite of the significant viscosity of the cytoplasmic medium, diffusion times are effectively tens of milliseconds [38], which is possibly one of the reasons evolution of these microbes has been towards minimally sized cells, on the scale of one micron [39].

The degree of semi-isolation in any given pond will depend on factors affecting advective transport, where currents are driven by thermal/gravity gradients, wind, or other environmental perturbations. Advection-diffusion modeling is required to account for concentration variation patterns in ponds [40,41].

The ultimate in horizontal heterogeneity would be for multiple ponds to be interconnected. If the ponds are identical in all respects, this may provide little advantage. However, if there are differences in size, shape, and especially contents, the opportunity arises for interactions which preserve the advantages of semi-isolation yet provide for the possibility of co-mingling of separately evolved molecules or independently evolved groups of primitive organisms. This possibility is further addressed in Section 3.7.

##### Foreshore

Another scenario, even more common, is a pond that is in recession due to lack of recharge, leaving the “bathtub ring” of deposits, mudflats, and possibly transient mini-ponds or meter-scale puddles. Solute deposition is common, as evidenced by salts and even organic residues. This area, technically known as the foreshore (or beach, or strand) may be of great importance in scenarios of both prebiotic chemical evolution and of biological evolution. 

The foreshore is composed of a littoral zone caused by wave action and by episodic flooding (direct rainfall, drainage area, streams, or rise in water table) and retreat (evaporative drying and seepage). It may also sometimes include a supralittoral zone, due to splash and spray under dynamic conditions, although this is less likely for ponds than for large bodies of water where the fetch of wind action is sufficient to form waves which break upon reaching the shore. 

**Figure 7:**
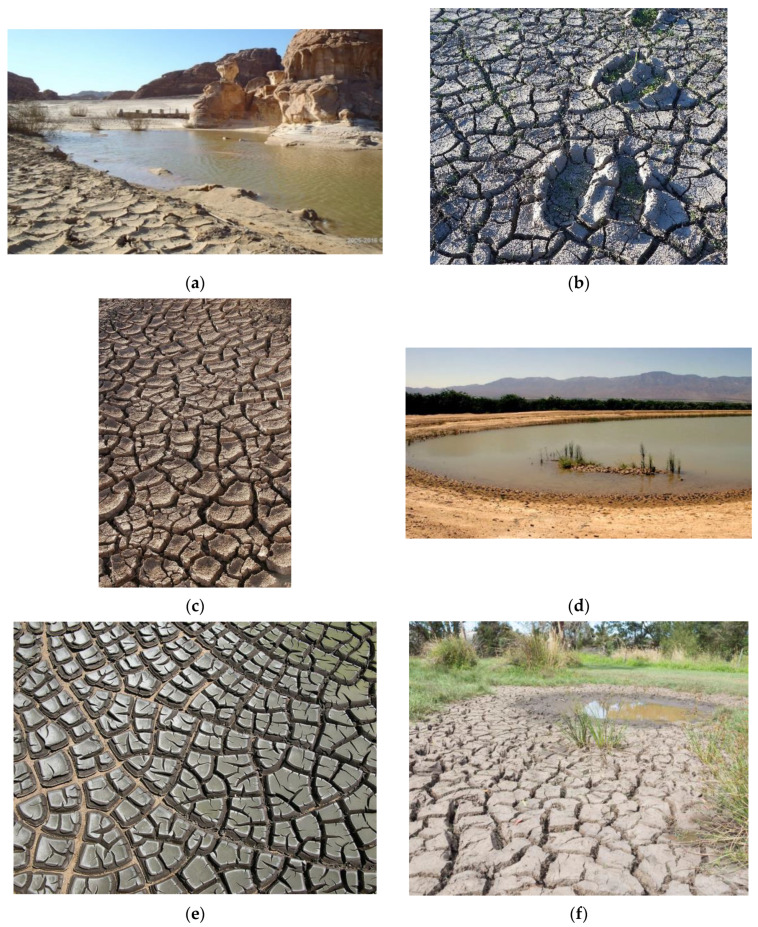
(**a**) Peeling mudcracks on pond. (Photo credit: www.discoversinai.net). (**b**) Mudcrack array (footprints for scale). (Photo credit: CSIRO). (**c**) Mudcrack peels. (Photo credit: by permission: © Tomas Castelazo, www.tomascastelazo.com, Wikimedia Commons). (**d**) Mudcracks on foreshore of pond. (Photo credit: © Gary Nafis, with permission; www.CaliforniaHerps.com). (**e**) Dried sludge (including organics from sewage plant; 0.5 m × 0.5 m). (Photo credit: Hannes Grobe/AWI). (**f**) Mudcracks and pond. (Photo credit: iStock standard license).

Mudcrack patterns can develop in locations where wet and dry conditions alternate, Figure 7. These crack networks are composed of irregular polygons whose perimeters channel and temporarily trap liquid. The cracks themselves typically have a linear scale of cm, but width in the mm scale and depth of several-mm. They can cover areas of meter scale, and the width of the polygons themselves is typically at the cm scale. Most importantly, they can transiently or permanently trap significant volumes of liquid. Example: for an average crack area of 3% coverage and average depth of 1 cm, the volume of liquid that could be infused in the cracks would be 300 mL per square meter of mudcrack network. Given that ponds can have several m^2^ of foreshore network, there can be multiple liters of liquid held in this zone which is highly exposed to atmospheric gases and solar insolation, as well as higher temperatures and repetitive wet–dry cycling.

It is instructive to consider how these mudcrack arrays could function as semi-isolated compartments. Each edge of each mudcrack polygon could hold ~1 cm^3^ of liquid (e.g., a 3 cm long crack, 2 mm wide and 1.5 cm deep). This may seem small. However, it is huge compared to the size of one bacterium, and even more so compared to a molecular replicator. It could accommodate 10^8^ to 10^10^ bacteria. For the RNA-world experiments using ribozymes, the concentrations are such that 10^13^ replicating molecules could be in this volume. Significant evolution could therefore occur, even in such minor volumes, and there are dozens to hundreds of such interstices in a typical mudcrack array.

It is also to be noticed in several panels of the Figure 7 that the topmost surfaces of the mudcrack polygons often each form shallow cups because of their upturned edges. This phenomenon, known as peeling mudcracks, provides yet another environment where pockets of liquid can be temporarily semi-isolated from other locations.

Mudcracks are the result of stresses which occur when minerals shrink under dehydration, such as certain clays (especially smectites such as montmorillonite) and many hydrated salts (such as sulfates and the many hydrates of Mg-chlorides). In addition to the physical heterogeneity in the horizontal plane, they are often graded physically and chemically, with the finest particles topmost and a less-cohesive matrix dominated by coarser grains at the bottom.

They can range in fragility, but often have durability against subsequent, repeated re-wetting. Typically, the junctions between cracks are not linear, but rather T-junctions of two cracks or Y-junctions of three cracks, which impede the reverse flow of liquid when the mudcrack pattern is temporarily flooded, although some patterns develop wider cracks which form linear drainage channels along multiple polygons.

Individual cracks will have more or less exposure to solar insolation during the progression of a diurnal cycle. Because they are near-planar at a gravitational equipotential, any given crack will experience a different solar profile, related to its azimuthal orientation and the latitude of its occurrence. With minor flooding, the material in most cracks may be relatively undisturbed, whereas with major flooding or atmospheric stimulation, their contents may be disturbed and redistributed back into the bulk of the pond.

Shallow ponds are typically endowed with a significant foreshore area. Even ponds initially bounded by scarps, such as cylindrical sinkholes or bowl-shaped ponds, may evolve to have more gently sloped shorelines from the combined results of active erosion, passive mass wasting, and soil liquefaction induced by wind loading or seismic shaking. We call this the “flared” shoreline case, which has many properties independent of the exact nature of the deeper bottom topography.

The multi-contoured foreshore annulus provides several functional advantages for origin of life scenarios. Reactions may be further augmented and speeded by unattenuated exposure to UV due to the shallow layers of liquid (except under salt crystallites or Fe-rich regolith grains).

The foreshore area enables repeated wet–dry cycles, possibly including mini-sequences of evaporites and other precipitates. It is also the loci where a pond first freezes and thaws—around its edges. These drying and freezing phenomena not only reduce the water activity, but they also enrich the concentration of the soluble ingredients in the residual liquid phase, and thereby promote increased reaction rates, especially for higher-order reactions. The formation reactions of nucleotides from their constituent nucleobases, ribose, and phosphate produce H_2_O and hence are favored under conditions that reduce water activity. Most importantly, the facilitation of the polymerization reactions necessary to proceed from nucleotides to RNA or DNA and from amino acids to proteins require dehydration reactions, which have been demonstrated to be enabled by wet–dry cycles [42,43,44,45] or also by freeze–thaw cycling [46,47,48,49,50,51]. In appropriate climates, such a pond may be subject to both wet–dry and freeze–thaw phenomena, thus strongly promoting the dehydration reactions for macromolecule polymerization by each of these distinct phenomena.

Ponds freeze from the shoreline inward and from the top downward. Ice formation causes excretion of solubilized constituents, which increases their concentration in the residual liquid. This provides ample opportunity for freeze–thaw phenomena, an area of increased interest as a medium for quasi-compartmentalization [51,52] as well as formation of nucleotide precursors, and their condensation into RNA oligomers [47,52].

The foreshore also produces successive segregation of certain components, according to their solubilities and in principle resulting in a multi-ring sequence of precipitated constituents. Gelation may be promoted and stabilized. Some sparingly soluble organics may be precipitated. 

There are thus cases where shallow gradients are also advantageous: (1) the pond’s beach has more area if forms on a very shallow slope; (2) intersecting zones to produce optimized concentrations that favor certain reactions and disfavor competing or wasteful reactions.

When the pond is “dying”, differential solubilities will separate out not just salts but also organics. Then, if it revives by addition of new water, there can be preferential mixing. Remobilization of salt evaporites on Earth are common, by meteoric and/or by marine waters, which results in complex relationships and modifications of the composition and distribution of those evaporites. This is likely to occur also with organics and clays, as well as salts.

**Figure 8:**
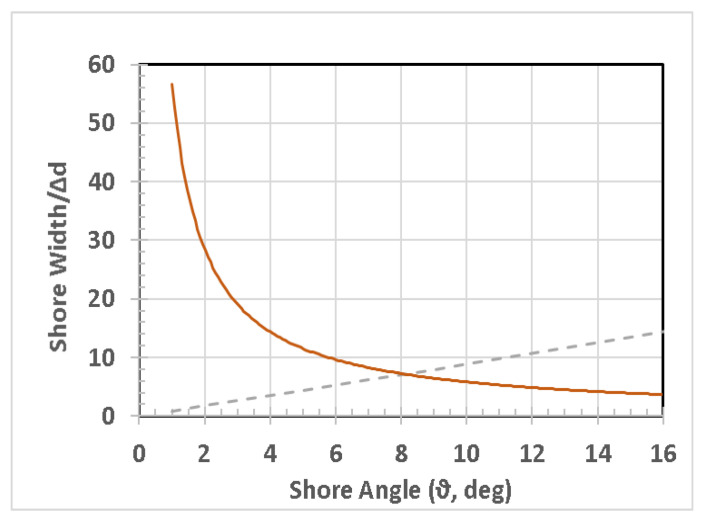
Expansion of foreshore for an incremental increase of water depth by Δd.

The area of foreshore is the product of its width and the pond’s circumference. In our “flared shoreline” example pond, Figure 4, the width is independent of the depth or even the detailed bottom shape of the pond. Such shallow-sloped shorelines are very common with bodies of all sizes, from ponds to lakes to seas and oceans. The relative change in shoreline width is dependent only on the local slope and is proportional to the cosecant of that slope angle. It is instructive to note from the plot in Figure 8 that for a 2 cm rise in water level, the shoreline width can change by one meter (i.e., 50-fold) if the slope is 1°. For a much steeper slope of 5°, the increase would still be over 10 times the change in water level. The amount of meteoric precipitation added to a pond includes not only the direct rainfall but also the runoff from its drainage basin, which can therefore be quite large. Foreshore width is also a function of transient disturbances, to the extent that winds and other meteorological phenomena have a sufficient strength and fetch across the pond to produce wave action. 

#### 3.3.3. Distributed Heterogeneities

The pond’s aqueous solution itself can contain not only those compounds which are soluble in water but also colloidal particulates which remain suspended due to currents and Brownian motion. Depending on relative concentrations, there may be formation of a sol, gel, or emulsion, each of which can cause further semi-isolation. 

Preferential physical adsorption and chemisorption of organic and inorganic ions onto certain mineral surfaces is yet another mechanism for beneficial semi-sequestration of potential reactants (or removal of superfluous or waste products). Mineral concentrations due to particle size effects, electrostatic charge, density, and other properties can also occur.

Another mechanism that promotes heterogeneity is co-precipitation, especially when amplified by increases in solute concentrations due to de-watering or by a rapid reaction that creates less soluble products. A geochemical example of the latter would be the encounter between two dense brines, one rich in CaCl_2_ and the other in MgSO_4_, resulting in the rapid precipitation of poorly soluble CaSO_4_ and a MgCl_2_-rich product brine, with ample availability of useful Mg^2+^. 

### 3.4. Temporal Changes

Over its lifetime, the pond will experience many different conditions, which are versions of heterogeneity and variations of semi-isolation with respect to time. These changes occur at many scales, from episodic to diurnal, to annual, and even to periods equivalent to the pond’s existence. Climate change occurs, even on simple planets. Diurnal thermal and relative humidity cycles can be quite extreme, especially at higher latitudes for planets with significant obliquity (spin axis tilt with respect to the normal to the orbital plane), and without a nearby moderating influence such as an ocean or large lake. 

Severe weather stimulating events, such as dust devils and stronger vortices, can significantly stir a small pond or portions of a large pond, to mix previously semi-isolated components and to restore it to more homogeneous conditions. Atmospheric microbursts can likewise cause disruption of heterogeneities and gradients. These events cannot be specifically forecast but can be common in many climates. 

We envision the pond as a potential palette of opportunities for localized, diverse activities within it, each semi-isolated but episodically coming together to produce new progress toward the OoL. Although these processes are fully subject to stochastic variability, the number of experiments that nature will conduct over the useful geological-scale lifetime of a planet or inhabitable moon has the potential to reduce the “highly improbable” to the “likely”. For example, if a planet such as Earth can have ~10^8^ candidate extant ponds, with a mean useful longevity per pond of 50 years, then during a 500 Myr span, there can be ~10^15^ individual experiments towards the invention of life!

Such a viewpoint treats the origin of life as a highly stochastic event. However, planet formation is also a highly stochastic event, with systematics that will only be discerned when we achieve a more comprehensive set of observations of other solar systems, and, after all, thermodynamics and chemical reactions are also just a macro-scale manifestation of an ensemble of stochastic events on the microscale. Life, through the shepherding actions of its enzymes, combined with controls over the concentrations of its internal reactants, overcomes much of this statistical variability in order to become more efficient at its primary function of reproduction.

### 3.5. Figures of Merit (FOM)

Each pond will be relatively unique, but there are broad characteristics that allow classification. Size and shape are some of these characteristics. Volumetrically larger ponds will be able to accommodate more molecules, and most importantly, more replicators. Since evolutionary advances are dependent to at least some extent on the quantity of replicators undergoing evolution, the larger ponds can potentially accommodate larger populations, as long as they are not nutrient limited. Larger-area ponds can provide diffusion-restricted semi-isolation among regions where different chemical evolution processes are underway, although if there are natural thermally driven circulation currents that are enabled, the degree of semi-isolation could be less substantial. All other factors being equal, larger ponds are longer-lasting. As they diminish in size, due to evaporation or seepage, they transition to becoming a member of the smaller population of ponds. If evaporative-driven, the concentration of important ingredients, such as nutrients, will increase.

The larger an individual pond, the greater chance that an organic-rich meteorite will fall into it, and the greater the chance that the energy of impact will not totally destroy that pond.

Since larger ponds generally have greater maximum depths, they are more suitable for attenuating UV, which is an advantage for survival of those molecules which can be destroyed by this radiation. However, when UV is valuable for its ability to provide energy to a reaction mixture, the attenuation may be counterproductive. Strong absorption can occur in the first mm or cm if there are effective UV absorbers in the water, including Fe^2+^, salts, or certain organic compounds, such as cyanide derivatives or soluble organic material from carbonaceous meteorites [37]. On the foreshore the water films become very thin at the high-water mark.

For the same alteration rates, smaller ponds can extract more nutrient elements from its subsurface contacts with soil or rock to more quickly achieve higher concentrations, because the ratio of contact area to pond volume decreases with diameter, D, and depth. This factor of increased interaction with an environment per unit volume is undoubtedly why prokaryotic cells generally optimize at the smallest possible size and adopt a spherical or somewhat spherical shape. For similar reasons, the foreshore area relative to the volume of a pond increases with smaller pond diameter. Because there are more small ponds than larger ones, the probability that any given meteorite falls into a small pond is higher than that it will fall into a large pond, but this is only a small factor because the small ponds provide smaller target area. However, the concentration of nutrients that can be extracted from the meteorite is greater (compare, for example, with the case that a meteorite falls into the ocean).

These various factors are summarized in Table 4, with an indication of which might be more favorable for ponds of various sizes. Open circles indicate less strong favorability for a particular size range than the filled circles.

**Figure 9:**
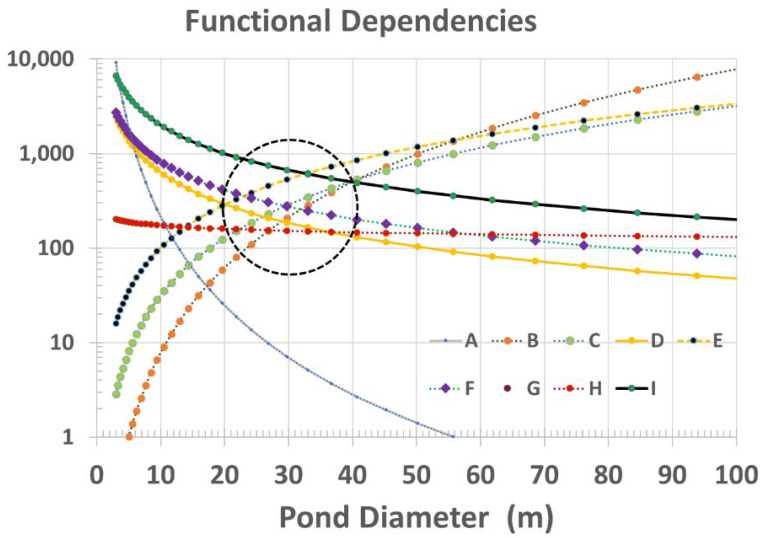
Functional dependencies of Figures of merit, with arbitrary weighting.

Because each of factors A through I can be relatively straight-forwardly be ascribed to various amounts and ratios of pond size, shape, and ISFD, we have calculated the functional dependence of each factor with respect to size for a conically shaped pond (depth ratio of α = 0.15 and rainfall precipitation of Δd = 2 cm). As plotted in Figure 9, each curve is given an arbitrary relative figure of merit (FOM) to illustrate the process. Where many curves overlap within a given size range, this is denoted by the overlain ellipsoidal outline. Clearly, for this example, smaller ponds, in the range of 30 m diameter, are overall more favorable than larger ponds. Although we believe this to ultimately be true in general, it is currently far too difficult to assign FOM scaling for several of these factors (relative to one another) without taking into account the particular hypothesized scenario for prebiotic chemical evolution and the specific environmental conditions on the planetary body of interest.

**Figure 10:**
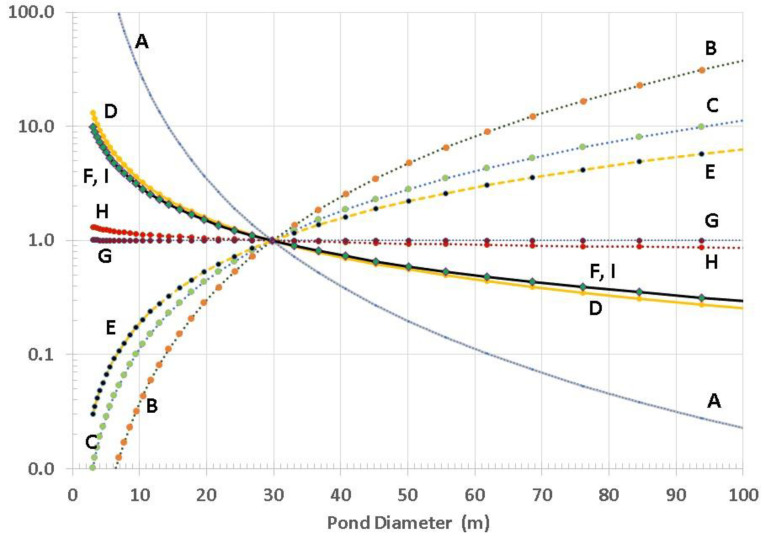
Functional dependencies (normalized to 1.0 at 30 m pond diameter, conical shape, α = 0.15). See Table 4 for key to curve labels.

Therefore, we have replotted these sensitivity curves with arbitrary normalization by adjustment of FOMs to create a relative value of 1.0 at a pond diameter of 30 m for each curve, as seen in Figure 10. Although arbitrary, this method provides a better means to inspect the relative sensitivity to pond size for each factor. This also clearly shows how factors G and H, although distinctly different parameters, are not particularly sensitive to pond size.

Factor G deserves some additional discussion. This factor relates the concentration of nutrients in the pond water to the area of the foreshore. Although smaller ponds can produce higher concentrations, they have smaller total area of foreshore (for the same Δd, e.g., rainfall or evaporation). We therefore calculate this factor as the product of foreshore area and concentrations of reactants in pond water. The result is independent of pond size because the foreshore area scales as D Δd, not D^2^.

Through evaporation, the foreshore areal deposits can build up to higher concentrations and undergo chemical reactions. Furthermore, there can be a hysteresis because the time for evaporation to form the mudcrack deposits of clay, silt and salt may be much longer than the time of re-flooding due to wave action or addition of water, and dissolution may be significantly hindered. Consolidated clays often do not readily re-disperse, depending on which exchangeable cations are involved in their initial amalgamation.

### 3.6. Smaller Is Better

As we have seen, a smaller body of water has many advantages over a larger body, partly because of its far higher incidence, but also due of several other key factors. Because the ratio of surface area contacting soil and rock to the bulk volume of the water body is higher for smaller ponds, it enables higher concentrations of extractable key elements within the finite time scale needed for aqueous alteration of the regolith in which it is in contact. This includes the CHNOPS group of elements, but also K and the metal elements that are also essential for life today, such as Mg, Ca, Fe, and many transition elements [53,54,55,56,57]. Although typically available at only trace levels (ppm), the first-row d-block elements in the Periodic Table provide various catalytic activities. Biology uses V, Mn, Fe, Co, Ni, Cu and Zn, although not Ti nor to any certain degree Cr [58]. Their bioavailability, or lack thereof, may be as limiting as when some of the key CHNOPS group elements are restricted. Only for elements which reach saturation equilibrium will this *not* be an important factor. 

As example, the contemporary ocean is well undersaturated for its major and minor soluble constituents, after eons of opportunity for equilibration with terrestrial minerals, and this is unlikely for the pond as well. However, even with the highly sophisticated and proactive capabilities of marine organisms to garner nutrients, there are major limitations on nutrient availability, especially with respect to P and N, which render ocean water habitable to a much lesser degree than many small lakes, ponds or swamps. When nutrient supplies are locally increased, eutrophication occurs, with marine algal blooms and their ecological consequences.

#### 3.6.1. Nutrients from the Bottom Surface

Often not included is the second part of Darwin’s musing about the “warm, little pond”. What he continued to say was, “pond with all sorts of ammonia and phosphoric salts, light, heat, electricity etcetera present, that a protein compound was chemically formed, ready to undergo still more complex changes”. [25]. He obviously knew that N and P were essential elements, and even knew that they needed to be in certain chemical forms to be effective as fertilizers. Realizing the continuity of life, he surmised that these same nutrients were required to enable the most primitive organisms to arise.

Based on the reducing nature of the Earth’s earliest environment, certain restrictions can be placed on the probable minerals that were common. Such studies [59] result in a significantly reduced set of such mineral compared to those extant today. However, in an extended study which takes into account other factors, such as near-surface hydrothermal systems from major impacts and photo-oxidation of transition metals by UV, it is concluded by the authors that key elements, including B, Mo, and P, will be present as minor or significant trace levels that can be become available for the origin of life [36]. This conclusion does not, of course, guarantee that all useful or critical trace elements will be bioavailable together in the same location under all conceivable environmental conditions.

Of the essential major elements, the soil minerals may supply N, P, and S. Nitrogen in minerals is very rare, but nitrates produced by lightning may be a component in soil [60]. Both P and S occur in many minerals. Some elements abundant in many common minerals include Mg, Fe, Na, K, and to a lesser extent P and S.

Beyond these, several elements needed as co-factors of key enzymes are normally present at only trace levels (ppm scale) in geologic materials. The majority of the key trace elements are the top row transition elements in the Periodic Table, which are to be found in approximately one-half or more of all enzymes [54]. Although the metallome fingerprint can have different relative abundances of these elements in different prokaryotes [61] and even vary significantly in a single species as a function of its environment and growth dynamics, it seems clear that early evolution of metabolic pathways before the directed synthesis of specific protein enzymes would have depended upon a variety of these elements. Aside from individual elements, there are the Fe-S clusters which are fundamental to electron transporting proteins such as the ferredoxins, and other metal-S clusters involving, for example, NiFeS in hydrogenase and MoFeS in nitrogenase. The residue of a metallome from 3.33 Ga carbonaceous residue in the Barberton indicates the early participation of Fe, V, Ni, As, Co, as well as modestly enriched Mn, Cu, Zr, but an absence of detectable Mo and Zn [57].

Over time, the pond medium will alter the minerals it is in contact with, which can liberate some of these key trace elements. The facility with which the pond can do so is affected not only by which minerals are available but also the temperature, time, Eh, salinity, and especially a low pH of the pond aqueous medium. Although the pH for H_2_O in contact with the usual mafic igneous minerals is in the direction of alkalinity, the pond itself can become temporarily acidic when major releases of acidic volatiles such as H_2_S, HCl, and SO_2_ occur during volcanic eruptions, especially if the S-gases can be further oxidized in the atmosphere by UV photolysis of H_2_O to produce strong oxidants such as -OH and O*, resulting in even stronger pond acidification by SO_3_ vapor and/or H_2_SO_3_ and H_2_SO_4_ aerosol. Once reactions are complete, the pond will revert to mild alkalinity, especially if carbonates have formed, until another significant release of magmatic volatiles occurs. Smaller ponds will respond more quickly and thoroughly to the influences of pH-lowering gases, aerosols, and rain than larger bodies of water because of the area/volume relationship.

If cyanide is available, such as provided by HCN from the atmosphere, there can be a capability to solubilize several relevant cations, including Cu and Zn. Smaller ponds can have an advantage in leaching out key elements to achieve higher concentrations than larger ones because their ratio of contact area with bottom minerals to the total volume of water scales with the inverse of diameter. 

On the basis of available concentrations, the extracted trace elements are limited by mass balance via the following relationship to the thickness, T_i_, of rock or soil particles that must be leached to achieve a useful concentration:

(3) $$T_{i}=[c_{i}m_{i}/w_{i})][1/(\varepsilon_{i}\rho )](V/A)$$

where c_i_ is the molar concentration of the element in pond water for the i_th_ species; m_i_ is its atomic mass; w_i_ is the fraction of mineral mass that is accounted for by the trace element (typically, ppm-scale); εi is the extraction efficiency (%); ρ is the mass density of the grain, and (V/A) is the ratio of the volume of pond water to the area of its contact with the soil. Extraction efficiency is a significant function of pH and temperature for many of these trace elements.

**Figure 11:**
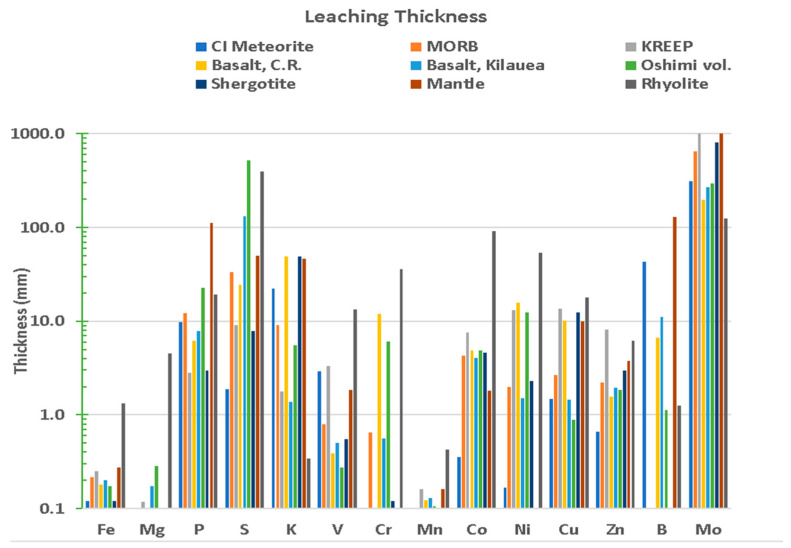
Thickness in mm for extraction of solubilized elements as a function of type of geologic material at base of pond to achieve a concentration of 1 mM for Fe, Mg, K, P, and S and 10 µM for all other elements.

For an example calculation, we set several parameters to common whole numbers to permit the reader to easily extrapolate their effects to any other values of interest. In Figure 11, we plot for several key elements the thicknesses that must be leached in a spherical cup-shaped pond to achieve nominal molar concentrations of 10 μM for trace elements, or 1 mM for elements whose concentrations are intrinsically higher or are needed in higher concentration (P, S, Fe, K, Mg). Other parameters chosen for this example are a pond diameter of 10 m; shallowness factor α of 0.1; an extraction efficiency (ε) of 50% and a solid particle density (ρ) of 3 g/cm^3^.

Achieving these extraction levels may, however, require acidic processing or higher oxygen levels than available in the early atmosphere. Some transition metal elements are more easily solubilized under reduced conditions, such as Fe^2+^, whereas others are more readily brought into solution under high Eh, such as Mn^4+^. In practice, the behavior of Mn in natural aqueous environments is dependent on physicochemical factors such as pH, redox potential (Eh), organic matter, specific conductance, and the presence of organic and inorganic complexes [62]. The same factors also need consideration with the other transition elements. The concentrations of boron are quite variable as a function of the source material, but can form a number of different minerals, the salts of which are quite soluble.

Results of calculations are plotted for the specific element concentrations for several different assumed materials, including mid-ocean ridge basalt (MORB) [63], several volcanic basalts [64], lunar KREEP [65], a Mars basaltic meteorite [66], Earth’s mantle and crust, and CI carbonaceous meteorites [67]. Based upon the ppm or weight % values for element concentrations in source material, more or less of these materials must be dissolved or leached. The chemical form of the extracted elements will be determined by several other factors, but as long as they are solvated, they will be potentially available to catalyze or to become incorporated in prebiotic reactions.

The figure is instructive in several ways. Several elements of odd atomic number (e.g., B, N, P, K, V, and Cu) are victims of the Oddo–Harkins rule that their solar system abundances are significantly lower than their neighbors having even numbers of protons, because of stability fields in nucleosynthesis. However, this is partly overcome by departures from these initial concentrations in the magmatically differentiated geologic materials which may have been present when potential macrobionts formed. It is seen that for many of these candidates, the leaching of only mm could provide useful levels of nutrients.

Phosphorus is an example of a potential difficulty, because its most common mineral form as apatite is not readily soluble [68,69,70,71]. Various solutions have been proposed, such as availability from the meteoritic mineral, schreibersite [71], or the ammoniated Mg phosphate, struvite [72]. Moreover, with modestly lower pH, it is possible to solubilize apatite [73]. It is also possible to reduce phosphate with Fe^2+^ [74] under hydrothermal conditions.

It may be surprising that sulfur, which is so ubiquitous and essential throughout our biosphere, is in fact a trace element in these terrestrial and Martian basalts, ranging from about 20 to 400 ppm, except for the Mars shergottite (~1200 ppm), the lunar KREEP analog (700 ppm) and in meteorites where it is at high concentrations of several weight per cent. It is instructive, however, to consider that the Martian global soil unit contains more than 20 times higher S than Shergotty, as sulfate, which is presumed to have been emplaced via the atmospheric route from volcanic exhalations as H_2_S and/or SO_2_, which become SO_3_ via interaction with photochemical products [75]. Although S is the essential chalcophile and is also siderophilic, it is a highly volatile element in both atomic and several molecules forms and is readily released to the atmosphere from magma. Early Earth and many exoplanets likely had abundant S available at critical times in their history. For our macrobiont, the main useful input of S may be via atmospheric interaction to scavenge the abundant sulfur species released from magma, as well as the S imported by meteoritic bombardment.

The release of Fe and Mg from aqueous alteration of the generally abundant primary igneous minerals may also be accompanied by the release of several other elements which are readily ionically substituted in those minerals. Those elements of biological interest can include Zn, V, and Cr from pyroxenes; Ni from olivines; and Mn from both. These will generally be available in solution as cations, although native Fe^0^ and Ni^0^ are abundant in many meteorites.

As seen, vanadium is at sufficiently high concentrations in basalts (~50 to 500 ppm) that only 1 mm depth of leaching may be sufficient to provide useful concentrations in this example pond. Other transition elements, such Co, Ni, Cu, and Zn, may require 1 cm or more alteration to achieve the 10 µM concentration postulated for this particular pond scenario. However, Zn is a relatively volatile element and, like S, may be provided by release from magma and then taken up by soil fines, as it has on Mars [76,77]. For Mo, which is often present at only sub-ppm levels, it could require an unrealistic alteration depth of one-third or more meter.

Potassium is generally abundant but can be recalcitrant to leaching because of being locked up in orthoclase and microcline feldspars, or as their illite clay weathering product.

Whether any specific pond can achieve significant levels of these elements by leaching within a time period significantly less than the survival time of the pond itself depends on the environmental conditions of pH and Eh, as well as the physical susceptibility of the minerals themselves, including their granularity and porosity. Fine-grained soil provides more porosity and surface area for alteration, but potentially allows more seepage from the pond. Shocked material (from impacts) and glass are more susceptible to rapid alteration. Some elements are more readily obtained from atmospheric gases even when available also in minerals.

Given that these elements may be needed to catalyze prebiotic reactions (whether acting alone rather than as a co-factor in a powerful protein-based enzyme), the functional strength of the macrobiont may benefit from aggressive alteration of country rock. It is also clear that the types of geologic materials (minerals and amorphous matter) can affect the time that the pond must interact with its environment to become the most capable of promoting the chemical pathways toward life. 

Achieving higher than these nominal concentrations can occur if, subsequent to extraction, the pond shrinks. Another pathway to higher concentrations is on the foreshore, where trapped overflow solution can undergo isolated evaporation and yet another method of concentration is possible if adsorptive clays, such as smectites, preferentially take up certain ions that can nonetheless participate in reactions with important intermediates.

**Figure 12:**
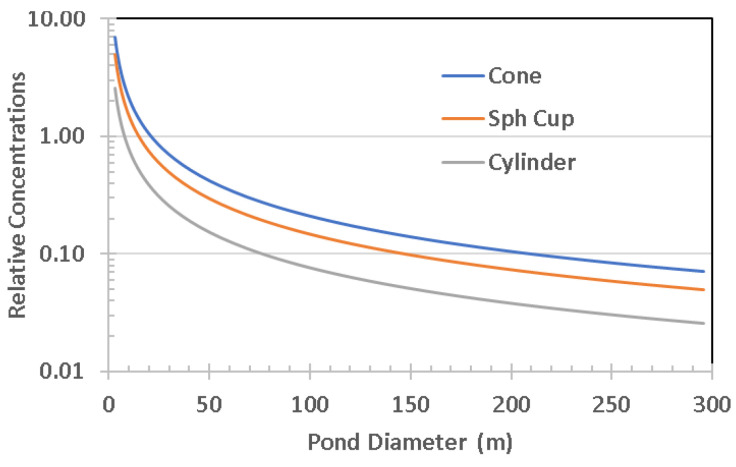
Relative concentrations in water of elements extracted from bottom soil and rock for different shapes (α = 0.15).

The bottom shape of the pond will affect the depth to which extraction must occur to reach useful concentrations of elements in the pond water. As seen in Figure 12, the conical shape has the greatest exposure to bottom area compared to total volume and a hemispherical pond has the least. Although the relative concentrations vary by a factor of 3.5 between these pond shapes, if normalized to the same value at some specific diameter, the curves would overlap to an extreme degree. Thus, the dependence on pond size is not as strongly dependent on shape as it is on diameter.

**Figure 13:**
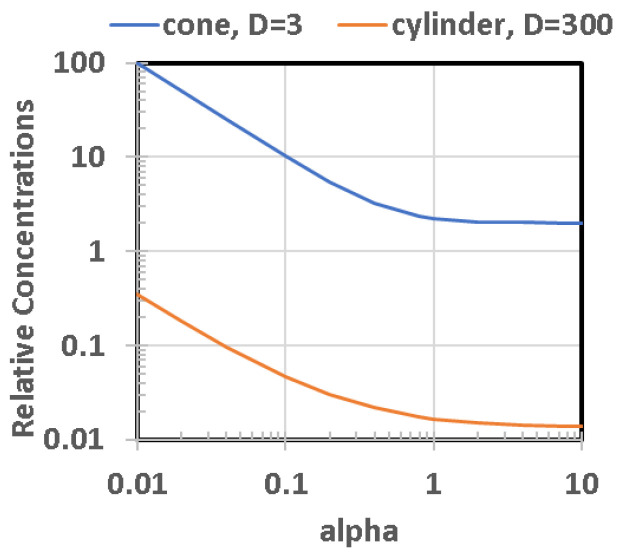
Concentration of nutrients for various maximum depths (alpha factor).

A shallower pond can achieve useful concentrations of dissolved elements with less alteration of bottom soil and rock. The sensitivity analysis of this is shown in Figure 13. The difference in concentration between the smallest cone-shaped and largest cylindrical-shaped pond is almost four orders of magnitude, and almost independent of the ratio of depth-to-diameter for α > 1.0. There is ~100× difference (arrows a and b) between these two extreme pond shapes, and the small conical pond is still superior by a factor of ~6 if it is deep compared to the large cylindrical pond (arrow c). 

#### 3.6.2. Nutrients from the Atmosphere

Compared to bottom surface area available for leaching, there will be a ratio of atmosphere to volume of the pond. In this case, the atmospheric constituents will be available in proportion to their concentration as dissolved gases. For gases which are inert with respect to the likely other contents of the pond, they will generally reach saturation much quicker than will those elements or molecules which must be extracted from the soil minerals. Nutrients which are provided by the atmosphere, such as gases containing C (CO, CO_2_, CH_4_, HCN), H (H_2_, and many others), N (N_2_, HCN, NO_x_, NH_3_), and S (H_2_S, SO_2_, SO_3_) are far easier to obtain than those which must be wrested from the minerals in which they are locked into the solid state. Moreover, certain gases, such as CO_2_, can dissolve at much higher concentration levels in H_2_O because of chemical interaction (at ~50x the concentration of dissolved N_2_ for the same partial pressure of each; in this case to form bicarbonate ion or carbonic acid). This is also similarly the case for HCN, SO_2_, SO_3_, NO_x_, and NH_3_, each of which readily form highly soluble salts, acids or bases.

Concentrations of dissolved gases in H_2_O are generally simply proportional to the partial pressure of the gas in the atmosphere, according to Henry’s Law, the constants for which are determined for a large array of gases [78]. However, in a pond these will be the concentrations at the air–water interface and can be less elsewhere depending on temperature gradients or the degree of advective pond mixing.

The availability of HCN for prebiotic organic syntheses has long been considered as plausible for a CO_2_ + N_2_ atmosphere which is subject to various energetic events, including lightning discharges, UV photochemistry, shocks from hypervelocity bolides [31,79], subaerial hydrothermal systems [80], or superflare coronal mass ejection events from the early active sun [81]. HCN can also be delivered exogenously, especially by comets [3,29]. Possibly particularly important is the formation of sodium ferrocyanide [82] as a temporary safe storehouse of cyanide for feedstock for prebiotic chemical evolution processes [83].

Another input of nutrients at the atmospheric interface can be regolith grains. For planetary bodies whose aeolian dynamic processes are strong and there exists a significant fine-grained fraction of particles in the topmost regolith, there can be inputs from saltation transport and also from settling after deflationary events such as regional dust storms and local dust devils. Silicate and salt-laden dust surfaces are easily wetted and therefore become irreversibly trapped in pond water, where they settle to the bottom since mineral densities typically span the range of 3 to 8 g/cm^3^, although soluble salts will dissolve and colloidal-sized insoluble grains may remain in suspension. These can be a very significant source of nutrients if the influx is high, but this influx depends strongly on the local weather conditions and the nature of the fine-grained component of the planetary regolith. 

An indirect benefit of a pond being shallow is that it will more vigorously respond to major transients, such as volcanic or impact-driven outgassing. Acidic components from the atmosphere will more quickly saturate a shallow pond than a deep one, and hence more quickly extract key elements from minerals susceptible to acidic alteration.

#### 3.6.3. Pond Temperature

Smaller ponds can become warmer (and colder) during a diurnal cycle, thereby speeding reaction rates during daytime. This could overcome the factor of their generally shorter lifetime. The smaller, more shallow ponds will be more susceptible to meteorological stirring by weather events, which can have degradable effects but at the same time may be indispensable for achieving the desired occasional mixing together of two or more semi-isolated areas.

**Figure 14:**
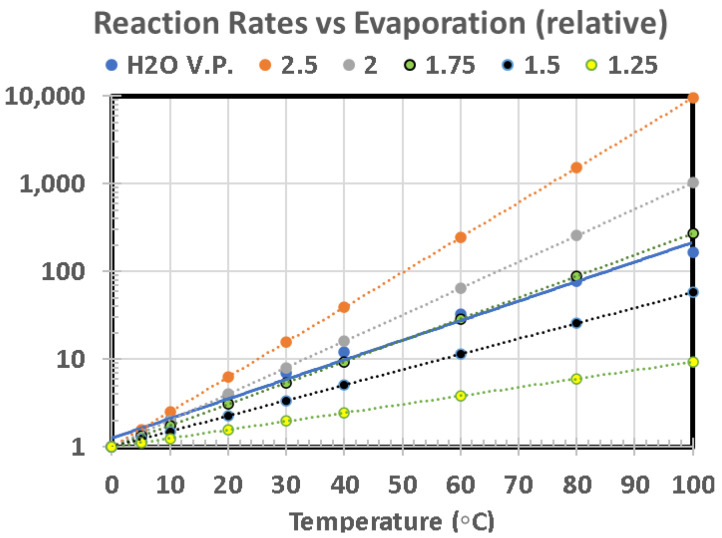
Plots of relative reaction rates for various reaction temperature dependencies (Q_10_), plus the vapor pressure curve of H_2_O (blue line). (All curves normalized to 1.0 at 0 °C temperature.).

The rate of chemical activity within the pond will be a function of temperature. By the general Q_10_ temperature coefficient per 10 °C increment, reaction rates increase by roughly a factor of 2 for many reactions, with a range of 1.5 to 5. For reactions with Q_10_~2, which correspond to a common activation energy of about 50 kJ/mol, a pond at 30 °C could evolve about an order of magnitude faster than one near freezing. However, the evaporation rate would also be rising because it is a direct function of the differential between the vapor pressure of the pond water (lower if briny) and relative humidity of the layer of atmosphere just above the pond’s surface. The vapor pressure (V.P.) rises exponentially with temperature. Normalizing V.P. and the Q_10_ temperature dependencies to 1.0 at 0 °C, we see in Figure 14 that the relative V.P. is increasing slower than the reaction rate for Q_10_ values below 1.75, but much faster for values above 2. This implies some potential advantage of higher temperatures when reproduction rates can be higher and hence progress toward chemical evolution could be faster without the same degree in evaporation. Evaporation will be slowed under a highly humid environment, a lack of wind, and any existence of an “oil slick” surface scum. Many factors can affect temperature, such as the potentially low albedo of the organic contents of the pond. An oil slick itself could produce a greenhouse effect.

Although the pond surface will be perpendicular to the local gravity vector, and hence experience a solar flux that is proportional to the cosine of the latitude for a spherical planetary body, the foreshore areas may have pro-solar or anti-solar slopes and hence experience a variety of temperatures. Although shallow-sloped foreshores will be larger, there can also be portions where there are much steeper, scarp-like shorelines. Shadowing would provide protected areas without direct solar insolation, resulting in somewhat lower temperatures and shelter from the direct effects of UV radiation. These may allow refugia for sensitive molecules, especially if they precipitate to provide additional self-shielding by outer layers.

#### 3.6.4. Pond and MB Longevity

The progress toward vigorous life in the macrobiont depends on many factors, not the least of which is the time available for evolution because of the finite lifetimes of small bodies of water. Although this is not necessarily the most important of the array of factors, it does set a hard limit on duration for evolutionary progress that can occur.

After input by rain, stream flow, or flooding, the pond can lose water out the top (evaporation) or into the ground. There is a fundamental difference in pond characteristics depending upon whether the H_2_O feedstock is being provided from above or below. Seepage is difficult to evaluate, although Pearce et al. have provided a model [40]. If fed by an artesian conduit, the availability of make-up water depends upon weather and climate conditions at a more remote location. Such a groundwater connection may be deleterious from the standpoint that it provides effectively a semi-infinite amount of water in which the needed constituents are ultimately dispersed, and thereby less favorable due to lower concentrations—unless the groundwater itself is an ample source of nutrients and redox couples.

Evaporative decline of the pond is an additional process. This is another important effect of area-to-volume ratios. Rainwater is nearly pure H_2_O, but groundwater can contain nutrients.

Calculating evaporation losses is straight-forward physics, depending on the surface temperature of the water and the partial pressure of H_2_O vapor in the atmospheric contact layer. However, the environmental factors affecting these parameters are quite variable and unpredictable because of uncertain weather patterns which change on both diurnal, annual, and climate timescales. Surface winds are an important factor because of its effect on the surface-interface vapor pressure. In conclusion, the longevity of any given pond is highly stochastic because it is driven by multiple factors, not the least of which are short- and long-term weather conditions. Based on terrestrial experience, it is reasonable to assume the duration of a pond is a function of size, and scales at time periods of years to centuries.

For a longer lifetime before evaporative drying to extinction, the pond will have a lower A_atmos_/V_water_ ratio for evaporation, which favors larger ponds, even lakes. However, evaporative losses also have significant advantages. As the pond dries, it creates a larger foreshore area and at the same time concentrates all the ingredients in the pond, thereby speeding up reaction rates. As it gets smaller, it may also transition from a thermally stratified pond to a more uniformly warmer one. Smaller ponds are also more susceptible to transient atmospheric disturbances, which can mix together quasi-separated constituents, including those hosted in the pond itself and that which resides on the foreshore.

There is an additional consequence that should be considered when assessing the value of the finite longevity of the typical pond. It is possible that a pond devolves into an intractable conglomeration of messy chemistry, which may not proceed along a path of prebiotic evolution. It could be better for the pond to become extinct and its ingredients dispersed. However, the geomorphologic pattern, whose shape is favorable as a catchment for forming a new pond, remains. This allows a rejuvenation, or rebirth as a new pMB, for a new opportunity for leading to life. Recycling of ponds provides a vast additional dimension by ~10^6^ to the probability space of achieving an origin of life that is adequate to seed a biosphere if we assume pond lifetimes are the order of hundreds of years, while the time available for the emergence of life on Earth has been judged to be a few hundred millions of year.

Besides loss of liquid H_2_O, there are other longevity limits to the activity of the pond MB. For example, in order to stay active, there needs to be a supply of energy. Assuming the capability for photosynthesis, even the simpler anoxygenic versions, does not arise early, the organisms will need to derive their energy as chemolithotrophs. Given that early Earth’s atmosphere is likely to have had a significant level of H_2_ in addition to CO_2_, like that of Mars [84,85,86], the exergonic metabolism of methanogenesis [87,88] would have continuously re-supplied the pond with energy and an independent carbon source. Alternatively, H_2_ could provide energy via reactions with sulfate, to the extent oxidized forms of S might be available.

The geologically brief longevity of ponds is overcome by larger bodies of water, such as lakes, great lakes, and oceans. Once a body of water is large enough, it can create significant areas of tidal flats, which are analogous to the pond foreshore and also create mudflats topography. However, a major difference is that an ocean is so large that when it floods and recedes, it causes extreme dilution of whatever advantageous new ingredients were formed in the flats by exposure to UV, wet–dry cycling, evaporative concentration, etc. These dilutions on the tidal ebb and flood time scale would be particularly disruptive.

### 3.7. Multiple Ponds

Not everything needs to occur in a single pond. Although we have made the case for a single overall setting, a scenario more favorable but less probable is a series of specifically different environments in a serial manner. In combination, a sequence of environments would still constitute the one overall Macrobiont because of their interconnectedness in either space or time. For example, separate ponds interconnected by streams or flood-level rainfall as one way in which multi-pot processes can be occasionally mixed together, has been suggested by both the Deamer group [45,89] and the Sutherland groups [17,90].

Neighboring ponds that are interconnected by saturated sediment provide a natural separation but also a potential purification mechanism, recognized as natural geochromatography [91]. They can also be interconnected in a temporal sense, through flooding or a rise of water table.

The Deamer group embraces hydrothermal settings, not sub-oceanic, but as hot springs around which wet–dry cycles can take place [42,89,92].

The Sutherland groups have studied cyanosulfidic pathways which can alternatively form the precursors to nucleotides, amino acids, and lipids from HCN and reduced S [93,94,95,96], depending on conditions. Their system also utilizes UV, Cu, Fe, P, Ca, and wet–dry cycles. 

The Carell group advocates a single location that is subject to changes in temperature, time, pH (4 to 9–10), wet–dry conditions and ingredient concentrations, to produce all four canonical nucleosides but also a number of other nucleosides with biological functions. Although this is all in a one-pot location, they assume the availability of simple cyano- and nitrile precursors as well as formic acid and Ni^0^ and Fe^0^ (e.g., meteoritic) [44], and ribose is assumed to be formed elsewhere. Key metabolites can also be produced using and Fe^0^ and Ni^0^ by selective reduction of CO_2_ to the acetate and pyruvate intermediates for the ancient reductive acetyl CoA (Wood–Ljungdahl) biosynthesis pathway [97].

Another advantage of multiple interconnected ponds is that it potentially can provide the equivalent of the classical “test-tube evolution experiment” [98,99] in which an organism (or ribozyme) is allowed to reproduce to near saturation (stationary phase), then an aliquot is transferred to a test tube of fresh nutrients, and the cycle is repeated multiple times. This has major advantages in speeding evolution because it selects a sample of highly evolved organisms and provides them with a fresh batch of nutrients and no waste products that could inhibit further exponential growth and evolution. Thus, _MB_C cells could get a fresh start by seeding other ponds having the essential ingredients already available. Moving aliquots into fresh media dilutes all the adverse conditions that accompany them (parasitic life; catalyst poisons; too many waste products; too little feedstock, including trace metals; etc.), and the _MB_C cells regain their population status because they can propagate, at the rate of 2^(t/g)^, where g is the average division time.

Although interconnected ponds can be less common than isolated ponds, especially in arid climates, they are by no means entirely rare. Streambeds on gentle slopes can bifurcate and meander, forming pools that are semi-isolated or even fully isolated during the dry season. This can happen anywhere, including in headwaters, but they are especially prominent as rivers debouch to form deltas. There, the braided streams and pools are ever-changing and provide a dynamic that could be favorable to the activities of macrobionts.

The cratering activity that is so predominate in the early history of planets provides natural topographic traps for water to form ponds. Large and especially energetic impacts send out ejecta blankets which include a multitude of secondary projectiles, each of which can create many more secondary craters. For example, the Zunil impact on Mars created ~10^7^ observable shallow (α~0.1) secondary craters (10–200 m diameter), and hydrodynamic codes predict a total of up to 10^9^ of such secondaries from the primary impact that created that 10 km diameter crater [100].

**Figure 15:**
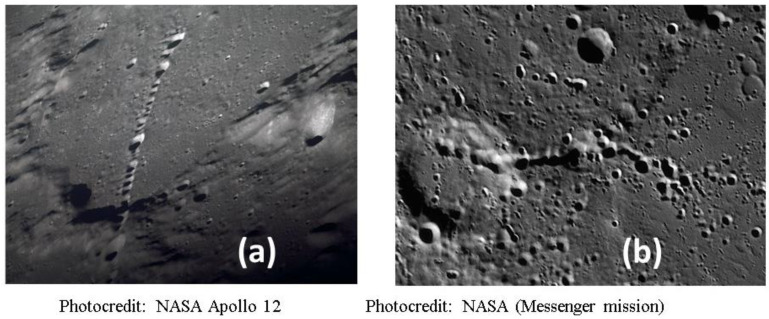
Crater chains provide possibility of interconnected ponds. (**a**) Lunar crater chain, Catena Davy; (**b**) crater chains on Mercury due to secondaries.

In Figure 15 are shown examples of multiple craters along a single lineament. Although relatively rare, they do exist and are known as catena in planetary geology, with several observed on Mars and other planetary bodies, so they would have occurred on early Earth as well, although all evidence of their past existence has since been erased.

This illuminates one of the characteristics of macrobionts. They no longer exist on an inhabited planet. They all disappear because virtually every pond on Earth is now already populated with a variety of organisms, including some which would consume the very organics which are needed to create the first primitive forms of life. However, ponds themselves and all their properties can be studied to reveal pathways for progress of abiotic chemical evolution having a high degree of plausibility.

## 4. Evolution within the Macrobiont

A system can also be tested for its ability to adapt its operation to specific multi-faceted hypothetical aberrations in the environment, including when one or more subsystems or components is not performing as original expected, due to failure or adverse, unexpected environments. This adaptability is termed “robustness” of an engineering system. In biology, it is the “plasticity”. As more rovers are developed and operated on the target planet, they can be outfitted with improvements from the “lessons learned” on the previous missions. Sometimes the improvements can be made onsite because of the programmability of the onboard computer. This is analogous to the “learning” behaviors of organisms. Other times, the improvements must be made on a subsequent mission, such as introducing better wheels less susceptible to damage when traversing strong, sharp rocks. With rovers, it is Lamarckian evolution. When microbes become more adapted to life on the planet of interest, it is by Darwinian evolution (survival selection of evolved progeny). 

A systems architecture also has the advantage that production can occur in parallel, because final assembly is the last step. Furthermore, subsystems and components can evolve independently and in parallel, which allows final assembly of an end-product which is superior in multiple ways.

Analogs of some life features may be applicable to evolution within the macrobiont. Horizontal gene transfer (HGT) is of the highest importance in the macrobiont if timely progress is to be made. Multiple plasmids could actually be better than forming a single genome, until all genes were highly optimized. Once there are two “winners” in terms of their independent capabilities, it is advantageous for them to fuse together so that the “whole” is superior. However, once a better version of one gene is invented, the ability to occasionally have cross-over shuffling makes sense as well. Thus, the early versions of mobile genetic elements such as plasmids and transposons, as shared via mechanisms analogous to bacterial transformation, transduction and perhaps even conjugation, could be taken advantage of by sub-par cells for much more rapid evolution if the population densities were high.

Given that cell densities can readily reach 10^9^ cells/mL for modern microbes, with doubling times being as fast as daily or even hourly, the number of generations per year for evolutionary refinements could be enormous. For example, even with a steady-state population of only 10^2^ actively dividing cells/mL in a small, shallow, conical pond of 10 m diameter and 1 m maximum depth, reproducing at a rate of only once per week but nutrient- and viability-limited such that cell turnover occurs but the total population does not increase, there could still be 10^12^ new generations in the short time period of 5 years. Or, in a 30 m diameter pond with an aspect ratio of 4:1 and a reproductive cell population of 10^5^ cells/mL that reproduces every 2 days for a remaining pond longevity of 50 years, the opportunities for evolution would be one million times higher, i.e., about 10^18^ generations. Experiments and models of RNA replicator evolution, assuming recycling, have demonstrated significant selection effects on short time scales [101]. As opposed to dormancy, cellular turnover due to competition for nutrients (e.g., events analogous to senescence and autophagy), cannibalistic predation, and/or bacterial programmed cell death [102] can enable evolution to proceed under oligotrophic conditions. Even isogenic populations of bacteria can have phenotypic heterogeneity, which allows a more rapid response triggered by quorum sensing [103]. Parasitism by molecular replicators that function as lytic phage could also produce the turnover that stimulates continued steady-state cellular evolution in a confined environment.

At contemporary natural mutation rates, this might yield minor progress. However, mutation and gene shuffling could have been at much higher rates in the macrobiont because of the lower fidelity of the genome replication process and the vagaries of an environment with chemical insults in particular and stronger selection pressures in general. As long as favorable mutants were not overcome by the waste product interference from poor-performing cells or protocells, evolutionary progress could be increasingly exponential with time.

Chromosome number in higher organisms makes sense for the same reasoning, and multiple alleles of the same gene in diploidy or polyploidy enable the opportunity to produce a more robust organism.

In addition to “Safety in Numbers”, there is also “Progress in Numbers”. For example, the explosive technological and scientific advances by humankind in just a few generations is because of the exponential growth in the number of engineers and scientists, and especially the directed and applied research between the two professions. Progress is made not just in one field or sub-discipline, but in dozens, even hundreds, of individual pathways down which no one person can go. Thus, although the technologies are totally different, we have progressed from hot-cathode vacuum tubes performing mostly analog functions to semiconductor solid-state integrated circuity performing analog but especially errorless digitally implemented functions. The semiconductor devices are also smaller, as well as more energy efficient, reliable, durable, safer, and much, much lower cost to produce. On the biological front, we have progressed in gene editing from radiation-, chemical-, or transposon-induced random mutations to the exquisite targeting of the CRISPR-Cas9 system. Not only is the latter far more specific, but it is fast, highly efficient, easy to implement, and low in cost.

These advances have been not only possible but realizable in time periods short compared to one human lifetime only because of the parallelism in advancements made possible by the widespread multiplicity of laboratories with trained technicians, discipline experts, and advanced technological equipment. Freely shared scientific results, combined with the inevitable diffusion of technological knowledge and advancements in spite of proprietary firewalls, avoid sluggishness in advances. Likewise, frequent HGT in the macrobiont speeds up the race to create a colonizing cell before the obsolescence and eventually demise of the MB itself.

### 4.1. Which First

In studies of the origin of life, there is the so-called “which first” conundrum. There are those who believe that metabolic pathways originated before genomic instruction sets and others who see compartments as the earliest breakthrough.

For controlled metabolic pathways and cycles, one needs a source of energy and especially the catalysts. Protein-based enzymes are powerful catalysts, but proteins themselves also can provide many other functions—they can produce structures; form motility organs such as flagella and pili; serve as chaperones to assist the folding of other proteins or shepherd key ions to desired locations (enzymes for which they are a co-factor for acquisition, or for disposal); regulate gene function in DNA; etc. Even as enzymes, there is an enormously broad range of catalytic styles, some of which may have originally been performed by the transition metal ions themselves, later superseded by improved versions of metalloenzymes, which not only have the key ion captured but have the 3D configuration necessary to position reactants with respect to the metal ion cofactor to facilitate the reaction.

The cell membrane is essential for the cell to be a self-contained entity, and, as has been often pointed out, it serves several other functions.

The instruction set, one of the sine qua nons of cellular reproduction and evolution that so dramatically characterizes living systems, is codified in linear, unbranched macromolecules, the DNA or RNA. There is no fundamental requirement for the linear feature, but there is no evidence of alternatives either, at least for our biosphere, i.e., LAWKI. RNA is especially multi-functional: ribozymes; ribosomes; tRNA, mRNA, microRNAs; siRNA; etc.

The fundamental question that has arisen is which of these three functions arose first? This conceptual juncture has many possible answers.

#### 4.1.1. RNA World

The first instantiation of life is widely considered to very likely have been a ribozyme which was autocatalytic for its own reproduction [5]. Whether it was RNA-based or some other replicating molecule, its appearance was surely the most significant event in the entire progression from inanimate matter to the bustling panoply of organisms in the biosphere we witness today. The invention of instruction-coded reproduction, whereby there could be among succeeding generations a version superior to its parent, was revolutionary because it enabled continued progress toward more and better advancements.

Although it is a matter of semantics, or taste, whether one defines the origin of Life as the first free-living cell or the first self-replicating encoded molecule, the latter was unequivocally a singular event, an inflection point in the evolution of the planetary body on which it occurred. Even if not initially RNA, it almost surely evolved to an RNA version [5] and hence the RNA World.

#### 4.1.2. Metabolism

**Figure 16:**
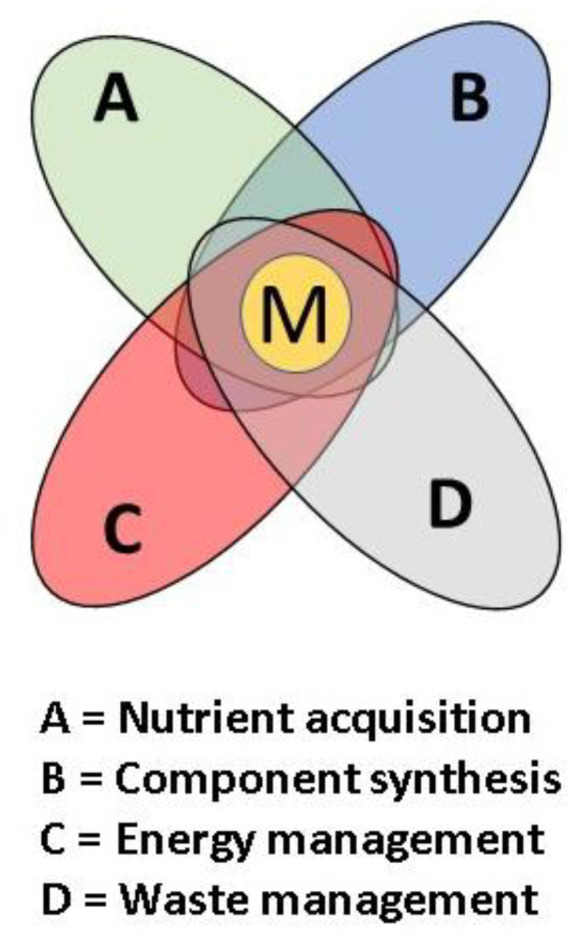
General metabolism includes several diverse and quasi-independent processes.

The term metabolism is a comprehensive concept that embraces all the chemical activity of the cell but in reality includes many different functions, each of which could be designated as a subsystem within our architectural block diagram. Here, we consider the four essential functions of synthesis, nutrient acquisition, energy acquisition, and waste management, Figure 16. Although each of these functions may initially partially occur within the macrobiont itself, the full development of an independent cell will require that much of this be under strict active, regulatory control.

Even in the RNA world, the prerequisites for the first RNA replicating molecule were the appropriate monomers, the activation of those monomers, and their subsequent polymerization according to a template. All these functions fit under this rubric of metabolism. Thus, a certain amount of “metabolism-first” is unavoidable, whether there were membranous materials present or not. Plausible prebiotic pathways for activation chemistry which combine these activities are under intensive investigation [104].

Life as we know it is based primarily on certain subsets of organic chemistry, although certain inorganic chemicals do play important roles (e.g., electrolytes, metal ion co-factors). A myriad of biochemical reactions is underway inside a typical microbe. Not only must it synthesize a variety of polymers, such as nucleic acids, polypeptides, and polysaccharides but also many of the monomeric building blocks and numerous other molecules.

The biological cell must synthesize all its critical components inside an existing, albeit expanding cell, before division can occur. This must take place without damaging the progenitor cell, including expansion of the cytoskeleton and one or two cell membranes, and in some cases also a cell wall. Although far more complicated overall, this enables an ultimate level of independence because the new cell is not reliant on some external system for its assembly. This self-assembly capability, however, is also difficult for an entity to achieve, and is perhaps the key criterion to distinguish between what is typically called the protocell and its next major step being to a cell which can take more optimum advantage of its environment, which we shall call the macrobiont cell (_MB_C).

##### Nutrient Acquisition

In order to conduct its other metabolic functions, the cell must acquire key nutrients. Those which are abundant can be obtained by consumption within the cell to create a diffusion gradient across the semi-permeable membrane. In modern cells, this is achieved by complex sensory and regulatory mechanisms operative at the membrane or periplasmic space. 

It is no small feat to detect, obtain, and regulate internal concentrations of trace elements needed for metalloprotein function, and there are specialized proteins for accomplishing these functions, usually each tuned to a specific element (e.g., Cu or Mn) [105]. In the earliest evolution, these sophistications must have been primitive or lacking, such that the macrobiont environment needed to enable a most primitive cell to develop in absence of it having these capabilities of the modern organisms. 

##### Energy Acquisition and Management

The ability to capture nascent energy from the environment, whether from photons or redox couples is another prime requirement for a cell. Furthermore, it must have the means to convert this energy to more useful forms, such as ATP and NADH, to drive the necessary reactions to achieve the other metabolic functions. 

This is also a fundamentally essential requirement for our planetary rover, which must use solar or nuclear energy to power its circuits, motors, and heaters, and must be able to store it electrochemically (battery), as well as convert it into its most useful forms (specific voltages). The overall system which delivers the rovers (rocket engines) also convert the chemical energy in propellants into heat and exhaust velocity, which are typically based on redox couples (bipropellants) or energy released during decomposition (monopropellant, analogous to fermentation). 

##### Waste Management

Another non-trivial problem facing any biotic assemblage is functioning in an environment in which other products are toxins or interfering clutter which do not contribute to the principal functions of metabolism. Some of these interferences are the result of essential metabolic pathways that are superfluous or useless end-products of essential reactions. Others simply originate from the environment or contemporaneous reactions that are non-productive to biotic function.

One way to dispose of unwanted products is catabolic reactions which break complex molecules down to simpler sub-units which may be useful or more easily discarded. Chemical digestive processes must be sufficiently selective, however, to avoid counteracting the primary functions of metabolism. Messy chemistry approaches must grapple with these challenges to an even greater degree.

Within the waste management function itself, there are several specific functions which can occur, including recycling (digestion to useful monomers or other smaller molecules), disposal (elimination by excreting to the pond or atmosphere), and neutralization (conversion to a non-interfering form). Some approaches to prebiotic synthesis have considered chemical flow reactors, for both streams/ponds scenarios [90] and suboceanic hydrothermal vents [106], and such approaches can provide opportunities for not only semi-continuous addition of key feedstocks but also to facilitate disposal of unwanted byproducts.

##### Ribosomes

Once the protocell evolves, will it consume or otherwise depress other distracting organics, especially the rogue RNA’s?

Ribosomes are the essential protein production factories of the cell, with rRNA as the ribozyme which catalyzes the linear assembly of protein molecules from amino acids specified by the tRNA anti-copy of an appropriate exon of DNA. Contemporary prokaryotic cells typically contain 10^3^ to 10^4^ ribosomes, accounting for up to one-fourth of the total mass [38]. Eukaryotic cells can contain 10^6^ to 10^7^, indicating a typical scaling of ribosome complement that is, to first order, linearly proportional to total mass, but also a function of reproduction rate. To support timely production of proteins, there must be a distributed manufacturing capability as evidenced by the quantity of independent protein production factories. We therefore envision that the protocell also contains the functions of rudimentary ribosomes, and the cC must surely have even more well-developed ribosomes, so as to be capable of adequate rates of protein production to survive the insults of the external environment and its fluctuations.

#### 4.1.3. Compartments

Compartmentalization is necessary for many reasons, the foremost of which is to contain biochemicals so that they are not diluted. It establishes an entity, the organism, which can pursue its own course of action and create its own internal milieu and eventually progeny with similar if not identical capabilities for survival, growth, and reproduction. Important is also the ability to control the movement of substances [15]—inward for needed nutrients, outward for waste products, and simple isolation to prevent the entry of substances which could poison catalysts, create unwanted reactions, or allow infection by parasites (e.g., phage). Another key advantage of compartmentalization, by vesicles or coacervates, is that it provides an additional level of semi-isolation as a concentration mechanism for needed feedstocks and for promoting proximity of interacting reactants and products. In contrast to the pond environment itself, this level of semi-isolation is on the microscale, and occurs as a multitude of independent cases, compared to the semi-isolated areas associated with the macro-scale properties of the pond itself. The MB system has created a whole new population of much smaller systems.

An outer envelope is fundamentally a 2-D construct, albeit one that folds back on itself. Adsorption or stronger bonding with this envelope by multiple entities creates a concentration mechanism which allows those entities to interact at a much higher rate than if they were randomly dispersed throughout the interior of the cell. It also provides the opportunity for those entities to interact preferentially with the outside environment.

Little wonder, then, that strong examples of the evolution of compartmentalization appear everywhere in the more complex eukaryotic lineage, with the advent of the double-membrane enveloped organelles, including the nucleus, mitochondria, chloroplasts, endoplasmic reticulum, and Golgi body. The vacuoles and lysosomes, as well as some other organelles, are also compartmentalized, although having only a single-membrane envelope. Complex multicellular organs in metazoans are generally enclosed by epithelial tissue, which is membranous and semi-isolating. The amniotic sac that protects the developing fetus is composed of two different membranes. Obviously, there are crucial multiple benefits of compartmentalization, as membranes, walls, and other barriers are incorporated over and over again in the biological hierarchy.

Yet, primitive precursor membranes in the macrobiont may be only fractionally effective at these functions. The fact that the pond macrobiont itself is a semi-isolated system, locked into a gravity trap, provides needed protection from the vicissitudes of the often-harsh dynamics of the global environment. As a refuge, it facilitates a more gradual evolution of membranes from amphiphile [15] or coacervate beginnings [107,108]. It also provides some diversity of mineral surfaces that may benefit prebiotic reactions via preferential physical adsorption or chemisorption of reactants, with the result of catalyzing favorable reactions. Examples include the montmorillonite clays [109,110]. 

A milestone would be when metabolism begins to synthesize the ingredients for organic membranes. If rudimentary metabolic use of vesicles allows improvements in their own production as well as advances in other functions, the system may develop not only synthesis capabilities but also processes to close gaps or repair mismatches. In this way, a completely enclosing geometric form, produced opportunistically, could result. The repair process may also enable the ability to “un-repair” locations to create specific passages and thus enhance selective permeability. This is needed not only for ingress of useful components but also for the egress of waste products and unneeded components.

Not only an encircling barrier must be constructed, but some semblance of internal physical organization may also be necessary for optimized replication via the process of growth and division. Improvements in efficiencies by having internal systematic arrangements can lead to a structural cytoskeleton, which is apparently ubiquitously present in all bacteria today [111] and also provides a myriad of possible external shapes which could optimize interactions for certain environments.

#### 4.1.4. Coevolution

As have others (e.g., [112]), we suggest the prospect of coevolution of these subsystems (metabolism, information, and membranes) and their other components. Somewhat analogous is the development of railroad transportation. Its three key components are the locomotive, the wagons it pulls, and the tracks it rides on. Which came first? The answer is none of the above, in the sense that dragging a container of items was early-on replaced by rolling, and then ultimately the wheel was invented (along with a rotary joint and its axle). Thus, carts and wagons were invented, pulled by people or by animal power, over natural or artificially improved paths and roads. With all the positive and negative topographic obstacles that could be encountered, the use of plateways was invented. Ultimately, rails were perfected, and the horses were replaced with locomotives. Both rails and locomotives underwent constant improvements, according to shape and composition for the rails, and progressive use of coal/steam engines, electricity/motors, and diesel fueled engines for locomotive power. Coevolution occurred routinely, often in fits and starts analogous to the apparent “punctuated evolution” of biological organisms. Rail transportation itself was eventually partially but not totally replaced by automobiles/highways, airplanes/airports, and rockets/spacecraft/rovers.

As advocated in various studies, the earliest form of compartmentalization could have been leaky coacervates [108], mineral surfaces [109], pores in hydrothermal vent chimneys [113,114], sulfide bubbles [115], vesicles from amphiphiles [6,15,51], and/or eventually lipid membranes [6,45,94]. Different versions may have played a role in rudimentary life before today’s versions arose. 

Yet, regardless whether there can be chemical evolution due to inanimate processes, it is difficult to consider something being “alive” without a genome, and difficult to envision a hardy lifeform without somehow maintaining together the critical components, such as within an appropriate envelope or compartment.

### 4.2. Macrobiont Stages

Any geologic environment is subject to change. Temporal variability can occur on all time scales, but the most relevant here is the timespan for the existence of the pond before it irreversibly loses its water to evaporation or seepage. Four stages in the progression toward a biosphere which must occur before the pond becomes extinct (irreversibly dry) are the following:

Stage I: A potential macrobiont (pMB) accumulated the ingredients needed for prebiotic chemical and physical evolution of essential molecular constituents. The molecular replicator arose, as well as primitive forms of semi-isolation and early versions of metabolic functions, which converted the pMB into an actual macrobiont.

**Figure 17:**
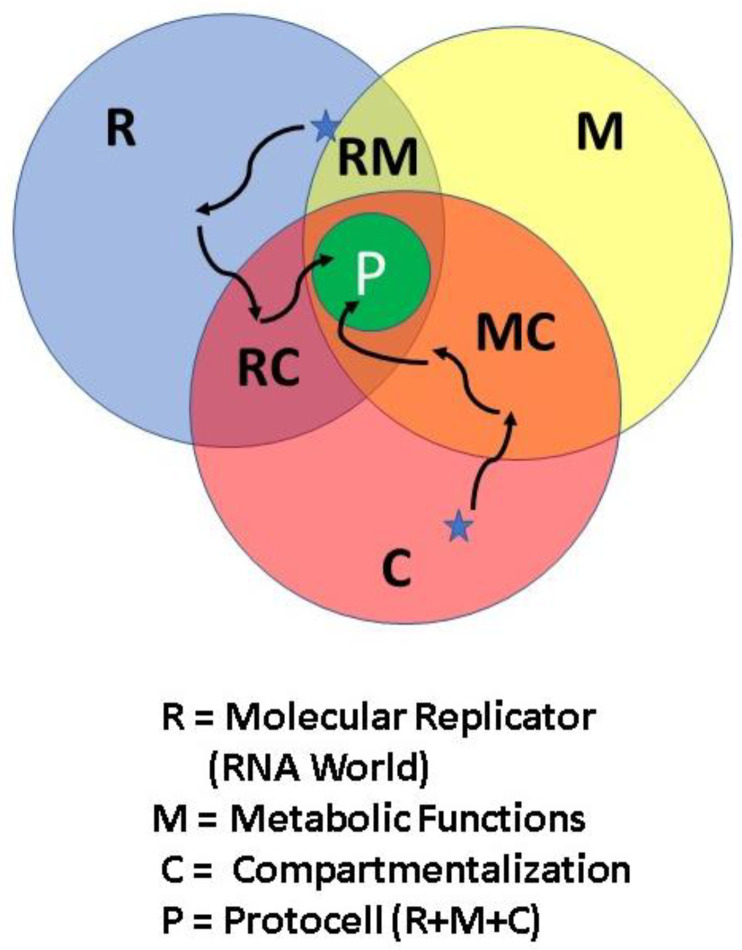
Arrows indicate one set of multiple possible pathways to the Protocell. (“star” indicates starting point).

Stage II: The three canonical ingredients evolved by some path and merged together to synergistically perform their functions and form what have been called “protocells,” Figure 17. However, although these protocells can exponentially speed up reactions and evolution of components, their propagation could be degradative. That is, successive generations could be deficient in reproducing their ancestors sufficiently accurately, and the future generations of any individual protocell could be less and less capable.

Stage III: A fully capable entity which faithfully produced equivalent progeny for many generations and therefore deserves to be termed a *cell* finally emerges. However, this cell is adapted to the MB environment, which is likely to be atypical compared to the environments external to the MB. This is a MB-adapted cell, the _MB_C.

Stage IV: Under various selection pressures and/or fully by chance, a version of a MB cell is sufficiently adapted to one or more environments commonly available outside the MB. This “colonizer cell” (cC) must create enough progeny that one or more escape the MB and begin the colonization of the planetary body, eventually leading to formation of a Biosphere which is robust due to its diversity. A successful breakout, through a groundwater conduit or by eolian transport of cCs in the foreshore, completes the “mission” of the macrobiont.

Individually, these stages are each major steps in comparison to the normal courses of events. They are aided by total time available but most likely also by the dynamism of the MB environment itself. Against this necessary timeline, the pond must survive and progress through all four stages so that the MB can survive an otherwise hostile world long enough to begin the formation of the rudimentary biosphere.

It should be noted that each hypothesized cell type is not a rigidly defined specific entity, but rather a class of organisms that envelops a range of individual capabilities and levels of development. Definition of these classes is, however, useful in that it identifies specific capabilities that must be achieved along the road to a global biosphere.

Otherwise, this attempt at creating a planetary biosphere may be self-abortive in most cases. Most will die out. Even most MBs may expire if they cannot go all the way to the cCell.

The quest for the so-called “minimal cell” number of genes does not take into account that a more primitive cell could have been far more inefficient, but simpler (“better is the enemy of good enough”).

The protocell is presumably not far enough developed to survive and flourish in the external environment. Probabilistically, it seems likely that most protocells will be destroyed before becoming a full MB cell, and that many _MB_Cs will run out of resources or perish before the first cC comes into existence, and successfully emigrates and proliferates.

However, this need not be daunting just because of being a multiple-probability sequence. As we showed in Section 3.4, on a typical planet like Earth (or Mars), there could be ~ 10^15^ opportunities to establish the rudiments of life. Even if this were only an RNA-world locked into a macrobiont, there still would be exponential-quantities of opportunities for progressing through each of the four stages we have envisioned. For example, if the progression from a simple pond to the occurrence of a molecular replicator is a one-in-a-million proposition, and the progression to a protocell consumes a one-in-ten thousand opportunity, there is still available a one-in-a-thousand opportunity for the protocell to produce the MB cell, and yet 100 ponds with opportunities for that form of life to become sufficiently endowed to be able to venture into the outside wide world. Against these possibilities is the realism that all this must be accomplished during the limited lifetimes of ponds.

This may still seem formidable, but the justification for allocating fewer opportunities for succeeding stages is the recognition that once the encoded molecular replicator is achieved, the progress toward a biosphere is enormously enhanced by the advent of the genetically preserved evolutionary history. Were it not for the fact that such evolution must occur in a Darwinian pseudo-random walk, the creation of the colonizer cell could be even sooner and more certain. Horizontal gene transfer is an enormously favorable factor in this regard, because it allows accumulation of the evolutionary achievements of countless individuals, without the negative effects of crowding by those whose special talents are countered by its other, less-favorable features.

#### Blurred Transitions

Within the maturing macrobiont, we anticipate “blurred transitions” between its stages, in the sense that it is a deep multi-step process to proceed from inanimate matter to ultimately achieve a fully functional cell that can make a living outside the MB. From the standpoint of LAWKI, in order to become qualified as a potential MB, a local environment must have liquid H_2_O; available CHNOPS and other nutrients; and one or more sources of energy. These are stringent but clear-cut minimal requirements and should allow identification of pMBs on early Earth, Mars, ocean worlds, or exoplanets.

It also allows identification of settings that do *not* qualify as pMBs for the origin of life. For example, if an environment is permanently too dry (lack of liquid H_2_O or warm ice) and/or is exposed to high fluxes of penetrating ionizing radiation—such as Mercury and the Moon.

At the other end of the pathway from pMB to a biosphere, there can be certainty only if the endpoint is achieved of producing a cC cell which can propagate in wider settings. If that cell is a marine organism, it need cope with only one general environment. If it is a terrestrially oriented organism, it may need to survive transport from one compatible environment to another, or temporal resilience between episodes of more clement conditions (e.g., rainfall in deserts).

In-between these two endpoints, all transitions will be blurry. For example, depending on the definition of life that one adopts, i.e., whether it is at the molecular level or is at the cellular level, the setting will no longer be a pMB, but will have progressed to a full macrobiont, hosting life. Although there is a plethora of definitions of life [116], the macrobiont is itself agnostic. Some definitions prefer operational specifications which are so broad as to include any self-reproductive entity (e.g., [117]) or which also embrace molecules but are more LAWKI-oriented, such as the putative NASA definition of life as “a self-sustaining chemical system capable of Darwinian evolution” reported by Joyce et al. [118]. Thus, when the RNA World, or some predecessor, arises in the MB and that is taken to be the “beginning of life”, is it necessary that a single step between non-living and living be identified?

Although the concept of “protocell” is variable [4,7], it nevertheless is considered by some as the onset of living entities [8]. Nevertheless, still for others, as we have noted previously, it is the first free living cell, which is the hallmark of the beginning of life (or, the biosphere). Still others focus on last universal common ancestor (LUCA) of bacteria and archaea as being the most seminal example of the root of life.

Is it even necessary to search for such a conclusion when what matters most is not when that first spark occurs, but rather when the first organism that can spread planet-wide first emerges from the MB. This is a clearly definable step. After that seminal event, the MB is actually no longer needed and will undoubtedly be ultimately destroyed by the natural processes that have obliterated the geologic record of the Hadean and early Archaean eons. For Earth, we have no justification in expecting to discover the actual original MB that achieved the full success of creating the first cC. However, it may be possible to find examples of pMBs as remnants from the early Earth or analogs on Mars.

A key attribute of a pond foreshore is that it provides one of the most important components of selection pressure correlated to the outside environments. In order for an _MB_C cell to evolve the competence to emigrate into the larger world, it must be capable of withstanding temporary exposure to desiccation and UV, especially if it is to be dispersed by aeolian activity. The foreshore provides a variable exposure between life-sustaining wetness and life-challenging dryness, repeatedly and over long time periods, thereby providing an ideal special environment for selecting for evolution of greater resistance to the debilitating or lethal effects of desiccation and UV exposure.

Another favorable factor, however, is that the ponds are not identical. None of their sizes, shapes, compositions, temperatures, and dynamic histories are ever exactly the same. This allows a multi-pronged exploration of probability space that vastly increases the chance that one or more ponds can accomplish what we know with certainty can be accomplished, i.e., the creation of a biosphere at least once on a planet whose nature was not necessarily the most optimal with respect to our current understanding of the task.

An ecological succession of _MB_C cells would occur as a result of being the cells with the greatest reproductive rate, given the available feedstocks, and thus would become the most abundant. This could arise by any of a number of capabilities, ranging from more timely or efficient nutrient acquisition to more or better ribosomes. However, this does not guarantee it would be the most likely strain to survive in the outside world. Rather, an evolved subset of the beached community, not of the central pond environment, could be the cell that became the cC cell, and subsequently the bottleneck ancestor that led to the LUCA and the biosphere we know today.

### 4.3. The Evolution Race

As the MB evolves, its biological make-up will change rather radically. So will its active biomass. When a replicating molecular entity comes into being and begins to evolve its efficiency at multiplying itself, the number of molecules of this type will increase dramatically and the relevant feedstock organics will decline somewhat.

Once this replicator achieves additional catalytic activities for promoting favorable proteins for metabolic processes and membrane synthesis (or enhanced adsorption to mineral surfaces), there will be additional mass under the influence of biological activity. That is, as abundant replicators mutate there will be selection effects for those which can promote the synthesis of beneficial products. Once a protocell emerges having sufficient competency to have long-term fidelity and effectivity, it will multiply rapidly until its necessary resources are depleted, or its wastes are suppressing, or both. At this point, the selection pressure will be for greater competency and perhaps augmented metabolic capabilities for more efficiency and the ability to tolerate the external environment with greater control over its own internal environment. The originally crude enzymes with rudimentary metal cofactors will become more specifically tailored to certain elements.

This MB cell will then multiply extensively, presumably even more rapidly. Perhaps it will evolve from an initially bulky version to a smaller, more tightly integrated cell, complete with ribosomes and perhaps the rudiments of metal chaperones to regulate their internal composition. With time, the _MB_C will become more and more refined as it consumes the remaining resources, including presumably also the less competent protocells. This will cause another ecosystem explosion, leading to saturation equivalent to stationary phase in a laboratory-provided medium, although most likely at not nearly as high a concentration because of the more likely case of a missing nutrient or a suppression by toxic or counterproductive products.

Once a cC arises, possibly in media that has experienced the foreshore conditions, it is only a matter of time until fertile external environments become colonized. With no competitors or parasites, the colonization can result in high population densities at each habitable location, and the biospheric mass itself will increase exponentially.

### 4.4. Systems Analysis Factors 

We now review some of the key components of systems thinking [119]. This provides a checklist of systems analysis characteristics that should be defined, considered, and quantitatively evaluated when possible.

Boundaries. These separate the system from its environment. For our case, it is the physical size and shape of the pond. Examples of figures of merit for these parameters have been evaluated.

Multiple interacting components. These are the subsystems defined in the block diagram. For the practicing systems engineer, the job is to take the overall fundamental performance requirements and derive the requirements for each subsystem that, taken together, enable the system to meet its top-level objectives. Once each subsystem engineer accepts the top-level requirements for her/his subsystem, they then derive more detailed requirements on a hierarchical basis, often penetrating down to the 4th or 5th level. As long as they are able to meet higher level requirements, a lower level one may be refined or changed to a different approach. 

As applied to the macrobiont, the primitive metabolic pathways and the substrates and enzymes enable a diversity that reflects these possibilities. Diversity of options is common in life. For example, life has invented at least six different metabolic pathways for reducing CO_2_ to organic feedstock, any one of which could be adequate for the task. All are very complex, involving multiple intermediate and enzymes, except for the acetyl-CoA reaction sequence, utilizing H_2_, which is simple, non-cyclic, exergonic, and is the only such pathway that occurs in both bacteria and archaea [120]. It has also been shown that mild hydrothermal alkaline conditions with various simple natural minerals as catalysts, including magnetite and greigite (Fe_3_S), promote this reaction [120,121]. 

Exchange of matter, energy, and information. These are key aspects of how subsystems accomplish their tasks and coordinate their activities for the functioning of the overall system. At the macrobiont level, the fluxes of organic molecules, CHNOPS nutrients, and catalytic elements each can be evaluated as a function of the range of plausible external environments.

Complexity is inevitable in large, highly capable systems performing tasks far from the natural order. Biology is one such system. Rockets are another, especially because a failure of the smallest part can result in disaster. However, with a systems architecture of the type outlined by the standard block diagram given above, the detailed implementation of any given subsystem can be changed if the interfaces and functional relationships with the other subsystems are not significantly changed. 

Different subsystems may utilize many of the same types of components that are generally available, although in different ways. For example, in spacecraft and rovers we have a multitude of Si and Ge chips; Cu wires; polymeric and ceramic insulators; Au coatings; Sn/Pb solder; and structural alloys of AlCu, FeCrNi, TiV; as well as composite structural materials that may include carbon fibers and metallic honeycomb. Most of these are represented in each subsystem. 

Technological advances in any of these components can be incorporated to improve the performance of one or more subsystems. The most important aspect is that these improvements can improve the overall system without major changes to the system itself, often as a “plug-to-play” substitution that produces major upgrades with minimal disruptive side-effects.

Analogously, the various cellular subsystems may each contain a variety of specialized proteins, RNAs, smaller heteroatom organic molecules, and cations. It would be a mistake to try to characterize a cell, or a macrobiont by these components alone, without recognizing the functions that compose the subsystems that are at the heart of how the system performs. However, these components can be “improved.” 

For example, DNA is a superior storehouse of genetic information compared to less-stable RNA. Metalloenzyme alternatives are widespread. Newfound access to rare W or Mo atoms, instead of Fe or V atoms as co-factors, can enable a variant or more efficient nitrogenase. The ability of ferredoxins to store electrons is facilitated by Fe_n_S_m_ clusters, with variants having different n and m values. The widespread hydrogenases have variants of Fe-only and NiFe co-factors. Ribosomes contain many components that are highly conserved across all three domains of life yet exhibit several salient differences [122].

Emergent properties are the result of the formation of the overall system and are not the properties of any individual part or subsystem. This is the case for the early evolution of the MB through stages I and II (prebiotic organic evolution and molecular replicators). However, we have also shown how the microbial cell itself is a system with nearly all the analogous subsystems as a highly engineered complex system. Once the MB enters stages III, IV and V, it is now a system-of-systems. 

Emergence is generally the result of feedbacks, which can be positive or negative, that alter the rate at which various components operate. Combined with the non-linearity of such actions, new capabilities and features can arise. Such changes can even involve tipping points, where the system changes in such a way that it cannot revert to its original state. Clearly, the evolution of living entities is an example of such activity.

Resilience is the property of a system that it remains in a stable condition in spite of perturbations caused by changes in its external environment. For the steps of prebiotic chemical evolution, once a promising set of pathways are found, the question is how robust they are to conditions other than the ones for which success was achieved. For engineered systems, there is not only parametric testing but also “stress testing” where environments beyond the range of those expected are also imposed to see what it takes to cause undesired behavior or failure of the system. This degree of challenging demonstrated prebiotic pathways may be somewhat premature, but eventually should not only be systematically tested but the results published so that they do not need to be repeated later. 

Static and dynamic are two top-level states of a system. A static system would have a fixed outcome, while a dynamic one adjusts to the changes that are imposed on the system. However, “static” does not necessarily mean non-operative. For example, the homeostasis that cells achieve by active control of the import and export of environmental constituents and cellular products assures survival and even efficient operation of the cellular metabolic and reproductive machinery. This is a dynamic process but gives the appearance of being static. The ability to also be “dynamic” to proactively achieve maximal “mission success” (e.g., growth and reproduction) is a hallmark of a highly successful system.

Evolution allows for changes in the system with time. The standard procedure for assembling a spacecraft is to first build and thoroughly test the subsystems or their components, before providing them to the process of building the spacecraft itself, the so-called Assembly Test and Launch Operations (ATLO) phase of a space project. As various building blocks are delivered to the ATLO team, they are integrated in a stepwise manner, with functional testing of each unit before proceeding to the next step. In this sense, a space hardware system is in final development over a period of ~one year, whether a rover or a rocket. Sometimes a missing subsystem or component is substituted by a proxy unit of lesser efficacy in order for the development process to proceed. Similarly, it should be expected that various subsystems of the macrobiont and its cellular constituents may not be ideal. Indeed, the protocell is conceived as a first attempt at cellular life. It may be lacking in many metabolic functions; it may be inefficient; it may need already-processed feedstocks; it may be large, and it may not be fully reproductive in the sense of internal enlargement and fission. If it is too lacking, that particular macrobiont may be doomed.

However, given that some tiny subset of protocells finally achieves greater capabilities, closer to modern organisms, the resulting _MB_C will be able to achieve a takeover that allows an acceleration of progress that facilitates rapid growth and further diversification with the greater likelihood of spinoff of a cC from adaptations to the foreshore environment. 

Just as with intelligent technological advancements, evolutionary progression generally can build on previous accomplishments. It is this phenomenon which allows a major increment in progression to be achieved in less time than the previous incremental improvement. 

As seen, the macrobiont itself begins as a single system but as a result of its successful evolution to develop independent cells becomes the system-of-systems. Life has long been considered an emergent property of matter, and the systems concept readily encompasses that feature. Life is different from most systems, however, because it creates copies of itself and therefore proliferates, almost without bound. Because the macrobiont cannot recreate itself, it is the obvious essential transition between two significantly different classes of systems: the living and the non-living.

## 5. Comparison with other Macrobionts

Many concepts for macrobionts have been put forth. The largest number can be categorized according to subaerial vs. suboceanic; hydrothermal vs. atmospheric-class temperatures; and meteorite-fueled vs. non-meteoritic. This 3 × 2 matrix create six different combinations, which we shall briefly visit. Although other settings and processes have been considered, they generally have not benefitted from as much analysis to date. As sometimes acknowledged, two or more of these possible settings may have existed on the early Earth. It may be possible that the outcomes form two different classes of settings were both successful, and then merged their genomes, or that one type of colonizer cell succeeded over the other because it was earlier, more efficient, or more aggressive and drove the other forms to extinction. It is prudent to pursue to the extent possible several candidate macrobiont settings because, for example, some planets may be so arid that ponds are unlikely, and/or magmatically subdued that deep water hydrothermal vents (one such setting) do not exist. At the opposite end of the scale, we now know that this solar system and presumably so many others host ocean worlds which are iced-over and airless, and hence do not provide surfaces compatible with the persistence of liquid water or trapping of atmospheric gases for any substantial length of time. Nevertheless, they may be habitable.

### 5.1. Subaerial Ponds in Hydrothermal Settings

A next step in the scenario of warm little ponds is to consider very hot little ponds, in the regime designated as hydrothermal (variously ascribed to source water at temperatures >50 °C or >100 °C). Such locations have been also widely suggested for early life [89,92,123,124]. The advantage of higher temperature is that it speeds up geochemical reactions, to more quickly penetrate deeper into the bottom soil, and enables reactions with higher activation energies, including serpentinization of indigenous mafic minerals to create H_2_ and potentially also CH_4_ (from CO_2_). Interactions with atmospheric N_2_ under such conditions can also lead to synthesis of HCN and HC_3_N [80].

Hydrothermal processing invariably leads to more efficient extraction of divalent cations (Mg, Ca) and transition trace elements (V to Zn). Hydrothermal processing of native rock is particularly efficient at producing deposits of Cu, Zn, Pb, and Ba, but it also can produce concentrations of Mo, Mn, Co, Ni, W, V, and B [125], all of which can have roles in biochemical processes. Finally, it may also enable certain organic chemical reactions, although one of the chief arguments against hydrothermal environments is that many important organic molecules are unstable at higher temperatures [126].

Geothermal environments, such as that at Yellowstone National Park (YNP), caused by near-surface reservoirs of magma can create spring-fed pools of water up to its boiling point (93 °C at the altitude of Yellowstone). In addition, there are geysers, fumaroles and mud pots, often co-existing in proximity with hot pools at otherwise isolated locations. Aside from the spectacular impression they create, such occurrences are currently exceedingly rare on planet Earth. Similar features currently active may be found at Rotorua (New Zealand), El Tatio (Chile), Dallol (Ethiopia), Beppu (Japan), Kamchatka (Russia), Haukadalur (Iceland), and a few volcanos. 

The total area of thermally anomalous surface in YNP, as measured by the far infrared ASTER instrument from orbit, is only 65 km^2^ [127], a tiny fraction of the park area. Only a portion of this heated area is wet, so this is an over-estimate of hydrothermal locales. The composition of heated waters varies from neutral chloride to acid sulfate, although it is interesting that boron concentrations [128] can be significantly enriched (to ~3 mM).

If we assume that the worldwide totality of surface hydrothermal activity is 10× the heated area of YNP, and even if there were 25× more active areas on the early Earth, the total fraction of today’s land area would be only ~10^−4^, or ~200 times less than the area of non-hydrothermal ponds available today. 

**Figure 18:**
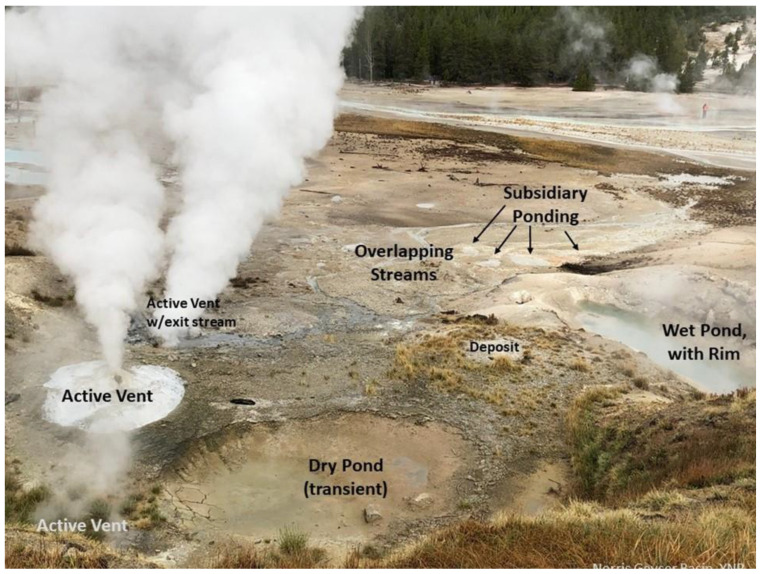
Mix of dry plus active ponds, and braided streams (Norris Geyser Basin, Yellowstone National Park). Photo credit: B. C. Clark.

As seen in Figure 18, there can be very favorable heterogeneities over short length scales in a subaerial hydrothermal setting. This is a relevant example of interconnected ponds, some connected by braided streams, as well as variable activity that spans the range form very wet to quickly dry. At the latitude of YNP, there is also freeze–thaw cycling that can occur during wintertime at locations where thermal activity is on the wane. Hydrothermal springs in sub-glacial or otherwise ice-rich conditions can occur over small spatial scales, such as in Iceland [129], and may serve to help preserve organics that would otherwise be subjected to thermolytic decomposition and aggressive hydrolysis, but must take place in restricted locations where both environments are juxtaposed.

Other hypotheses have favored ponds on the flanks of active volcanoes, taking advantage of their volatile emissions, hydrothermal locations, fumaroles, etc., as well as the sloping terrain leading to other ponds and ultimately to the ocean [89]. Similarly, relatively rare volcanic islands poking above a global ocean to provide WLPs has also been championed [12].

### 5.2. The Suboceanic Hydrothermal Vent

The seafloor hydrothermal vent (HTV) systems support a mini-ecosphere of organisms ranging from bacteria in tubeworms to shellfish and higher fauna, the food chain of which is supported by the abundance of dissolved elements and molecules in useful redox states pouring out of their “chimneys.” From a physicochemical standpoint, the chimneys are a type of a chemical garden, with a complex interplay of a plume of mineral-rich high temperature liquid precipitating in cold seawater, requiring a complex analysis by interdisciplinary means in the emerging field of chemobrionics [113]. For many reasons, as recently summarized [130], this is considered by many scientists as a strong potential locale for an origin of life. At the same time, there are problems with this scenario, some of which are the same as for the older idea of a “primordial soup” for the entire ocean.

Foremost is the dilution problem, i.e., that even though the density of ingredients is high in the interiors and at the mouths of the smokers, and in the immediate vicinity, those concentrations fall extremely rapidly with distance as these waters quickly mix with the ocean at large, aided by the currents and turbulence they create. An answer to this is to suppose that the OoL occurs within the pores of the chimney walls [130]. Or, that there are iron sulfide bubbles created from lower temperature seepage which would encapsulate ingredients for their further reaction [115].

An analyzed solution offered to this problem is if the relevant prebiotic reactions occur in pores in the affected rock material whereby accumulation of molecules can occur as a result of strong thermal gradients, and the pores provide not only an enhanced concentration and acceptable thermal environment but also a degree of compartmentation prior to the advent of membranes and active transport processes [114].

The vastness of the ocean also creates dilution problems for any exogeneous sources of organics and other matter to an origin of life there. The contribution of organics from interplanetary dust particles and meteorites can be significant when integrated over time, taking into account the much higher flux onto the early Earth [26]. However, when diluted throughout the ~3.7 km average depth of the ocean or its primordial predecessor, and allowing for various degradative processes, it is questionable whether they could achieve a useful accumulation of amino acids or other important organic precursors.

Another problem is that the even higher temperatures (up to 400 °C for black smokers; ~250 °C for white smokers) render many organic compounds unstable [126], preventing the full extent of prebiotic chemistry which must occur. Biologically relevant compounds, such as amino acids and some heterocyclic bases are found in certain types of meteorites [27], providing evidence of abiotic organic evolution at lower temperatures. In comparison, there are still many gaps in demonstrating the syntheses of critical components, including amino acids, nucleotides, and lipids by processes that could occur in hydrothermal vent systems [90], with the exception of a recent discovery of tryptophan [131].

Underwater, there is no opportunity for wet–dry or freeze–thaw cycling to promote the dehydration reactions needed to form protein and nucleic acid polymers. A colder early Earth [132,133], with more extensive polar caps, would provide the ice–water interface that could promote freeze–thaw conditions, unless the HTVs are simply too far away to supply key ingredients at useful concentrations. Ocean worlds such as Europa and Enceladus have similar considerations.

Both the MB pond and the MB seafloor hydrothermal vent have the problem that the cell which arises will be tuned to the environment in which it arises. This _MB_C cell must evolve to a colonizer-capable cell compatible with the wider planetary environment, which in the HTV case is the ocean itself. If the primordial ocean was poor in some nutrients, as is the contemporaneous ocean, there could be a selection pressure toward photosynthesis. On the other hand, once a cC cell did arise in the ocean, it could become a global entity, whereas land-based organisms face a multitude of different environments with which to eventually cope.

Compared to the surface ponds, these suboceanic hydrothermal vents are rarer. Indeed, they are so rare that it has been proposed that they be considered unique protected sites so that economic exploitation of their high metal content can be blocked under the auspices of the International Seabed Authority to prevent deep sea mining at these locations [134]. 

There has also been a growing emphasis on the alkaline, low-temperature white smokers, such as the Lost City field, as a more probable locale for the OoL [130,135], and these are much rarer than the black smoker hydrothermal fields. Indeed, the Lost City location has been singled out for highest priority protection against commercial exploitation [136].

Others have criticized the hypothesis of an alkaline environment as being one not conducive to prebiotic chemical evolution [137].

Seafloor hydrothermal systems undergo cyclic variations in temperature and vent fluid composition in response to magmatic episodes. The activity of the chimneys themselves can be short-lived, on the order of decades or less. However, it has been shown by radiometric dating that the serpentinization process in the Lost City peridotite mound has been underway for at least 30 kyr, and possibly could persist for millions of years [138]. Moreover, it is postulated that there are “billions” of “membranous compartments” produced over the lifetime of a single HTV system, such that “life’s emergence appears inevitable” [135].

Plate tectonic activity on the early Earth may have been weak or absent [139], which is relevant to the debate over whether the continents formed early or did not appear until long after life began. Vents occur at both diverging and converging plate boundaries. For today’s activity levels, an estimate for all the active vents worldwide is a total area of ~50 km^2^ [134]. This is a generous estimate in the sense that it assumes a 30 m diameter for each vent area, which accounts for its span of influence but is a much larger area than for the chimney sites themselves. Thus, today, pond area is >50,000 times more available than the HTV locations. If instead of area, one considers just the number of ponds versus the estimated number of suboceanic HTVs active today (5000 [134]), there are ~ one million more ponds in the size range of 10 to 100 m diameter than HTVs, and ~10 million more for our nominal 3 to 300 m diameter ponds. If plate tectonics was not yet active to form continents, yet the ocean existed, there would have been less land, if any at all, upon which ponds could form, other than volcanic islands. However, there would also have been much less hydrothermal vent area. 

Because the actual source of the uniquely vast amounts of H_2_O at the surface of the Earth is unproven, it also cannot be assumed that the ocean as we know it today fully existed at the time of the first fully successful MB. If our hydrosphere was formed by outgassing, the environment would have been extremely hot, but perhaps analogous to a MB scenario similar to today’s concept of HTVs. If the ocean was mainly delivered from successive impacts by water-rich bolides, such as comets, it would require a vast number of very large comets to do so (e.g., about 10,000 even if the average were the largest size estimated for the Chicxulub impactor, ~80 km). These impacts would be punctuated in time, but each impact could wreak havoc on a global scale.

However, as the natural lifetime of a pond macrobiont would be short compared to the mean time between local impacts, there would be ample opportunity for a successful MB to produce colonizing cells even during bombardment that seems frequent on the geological time scale. Moreover, although the Chicxulub impact may have destroyed the majority of life forms extant at that time, it did not extinguish all microbial life and other hardy or protected life. In periods of subsequent stochastically extra-intensive bombardment, the global environment may have temporarily changed so much that surviving microbes may have had to adapt to higher temperatures in the surface environment, or found refugia in subsurface hydrothermal reservoirs or subaqueous HTVs, resulting in an evolutionary bottleneck through which only organisms compatible with high temperatures could survive and propagate [140,141]. 

One line of evidence favoring a hydrothermal origin of life is the characterization of the metabolic capabilities of the LUCA organism. In a phylogenetic approach that identified 355 proteins that were “probably present in LUCA”, that study [142] implicated an organism which was thermophilic; chemolithoautotrophic using H_2_ to reduce CO_2_; N-fixing; did use FeS and transition element co-factors, including rare MoCo and Se; and was also equipped with rotor-stator genes. Nitrogen fixation would not seem to be necessary given that bioavailable N is already present in vents (NH^4+^ in high-temperature fluid and NO^3−^ in lower-T fluid [143]). Surprisingly, the impressive capabilities of this putative cell are not reflected in enzymes for biosynthesis of key metabolites, such as most amino acids, some nucleosides, and various cofactors, implying that these were subject to horizontal gene transfer or that the substrates were readily available in the environment [142]. 

A typical counter to these types of findings is that LUCA is not necessarily representative of the first free-living cell, which may have been more primitive. In particular, our colonizer cell, cC, may not have needed capabilities such as N_2_-fixation (and attendant significant concentrations of sparse Mo) or flagellum-equipped taxis responses. In addition to the possibility of major impact events causing a new selection pressure [140] against surface-dwelling biota, there could have been other environmental changes with a similar effect.

Findings from evolutionary-rate analysis of Yellowstone microbial communities are that photosynthetic organisms experienced evolutionary bottlenecks that thermophilic chemotroph organisms did not [144]. This is ascribed to episodic lava flows and glaciations which interrupted surface-dwelling organisms while the chemotrophs survived in networks of sub-surface hydrothermal channels. In analogy, after life got its global start, proliferated, and adapted to a variety of habitats, if global-scale volcanic resurfacing and/or climate change leading to an early version of snowball earth [132] occurred, the bottleneck refugia may have been subsurface or suboceanic hydrothermal systems, and/or impact crater hydrothermal lakes, where only thermophiles would survive and proliferate.

### 5.3. Impact Crater Hydrothermal Lakes

The high population density of impact craters on the Moon is indicative of the bombardment the early Earth was also subjected to. Currently controversial is whether there was a gradual decrease in crater formation rate or there was an episode of temporarily increased levels, the so-called late heavy bombardment. Large craters are evidence of impacts by hypervelocity bolides whose kinetic energy would have been partitioned into not only mechanical disruption of the surface but also heat. The cratering record of large impacts on the Moon, Mars, and Mercury is clear evidence that there was an abundance (thousands, or more?) of formations of craters on Earth sufficiently hot that they could be filled with water by melting of permafrost ice, or by subsequent meteorological precipitation over the available watershed. Such crater lakes would have provided both subaerial and buried hydrothermal regimes for durations that would depend on the energetics of the impactor (size and velocity) but could continue for 10^3^ to 10^6^ years for impacts producing crater diameters of ~5 to 200 km [145] as the thermal energy and/or water supply slowly dissipated. 

These crater lakes have been championed as candidates for the OoL by a number of researchers because of their favorable properties and inevitable past existence, e.g., [145,146]. Only for the larger craters, of course, would the amount and burial depth of heat deposited have been sufficient to create a long-lived, high temperature system. Therefore, the subsequent crater-filling hydrothermal lake would have been far larger than the “ponds” envisioned above. However, the secondary craters and surrounding hot springs that would be created could result in many pond settings derived from the impact itself.

The indications that the origin of the Earth’s biosphere is ancient [147,148] and suspiciously close to the time when the heavy bombardment was drawing to a close lend credibility to the crater lake hypothesis. However, the criteria analyzed and discussed in Section 3 above suggest that the highly abundant smaller bodies of water may have been the more likely setting for the first fully successful macrobiont. Such smaller ponds or hot-spring pools may also have flourished for a finite duration following each major impact. Magmatic sources can be longer-lived, compared to the hydrothermal character of the impactor crater lake, which will decay irreversibly with time. 

### 5.4. Meteorite-Fueled Macrobionts

During the late-stage accretion of the early Earth critical precursor organics were delivered by carbonaceous meteorites and the even more organically enriched comets. Although the amount of organic material that could be added during the later stages of bombardment could have been large [26], there is a “double-dilution” problem for this source in general [3]. The first aspect of dilution is the dispersal when large impactors at hypervelocity explode upon contact with the surface, and their ingredients are distributed through some depth of regolith, or worse, throughout the ocean. The other dilution factor is because the bombardment is spread out over a significant fraction of geologic time, the probability of timely occurrences of multiple independent impacts at any given location is very small. Those compounds which are susceptible to degradation due to interaction with atmospheric gases, water, metamorphic heat, ionizing radiation, and other disrupting factors will not be available in useful form indefinitely.

For this reason, one approach is to consider rare, stochastic events, such as a scenario that one of these bolides, after being slowed, lands into a small body of water. Thus, there is a comet pond scenario for creating a macrobiont for the origin of life [1,2,3]. This is a very low probability event if all comets are as fragile as most, because they generally ablate severely or explode before they can be sufficiently slowed before impact. However, if the indications are correct of unusually richness in organics, especially for cyanogens, then a comet pond macrobiont may be uniquely suitable for prebiotic evolution [3]. Moreover, a slowed cometary body needs not land in a pond, but rather could create its own pond by its high content of H_2_O ice.

Another hypothesis is that over time, many individual carbonaceous chondrites landed in natural ponds rather than on bare land [40]. These provided the starting constituents, including a variety of amino acids [28,149] and membrane-forming amphiphiles [15,150]. An advantage of this proposal is that it is amenable to laboratory analysis because of the availability of the meteoritic starting material.

### 5.5. Other Planets

Mars is sufficiently like Earth that the small pond is also a good candidate for the macrobiont on that planet. Indeed, for several reasons it might be even more suitable for this pond scenario. This is not necessarily the case for all the planets in our Solar System, and, in general, in the universe. There may be many, or even a majority of cases that are sufficiently different from our inner planets that one of these other scenarios may be even more likely to have provided the macrobionts from which a biosphere eventually emerged.

Not only would an exoplanet need H_2_O to facilitate an origin of life as we currently understand it, but that H_2_O would need to be in the liquid state. This is an indirect but key constraint on the need for an atmosphere, at a significant pressure. Because the vapor pressure of H_2_O is a strong function of absolute temperature, whereas the transition to the solid state (i.e., ice) occurs at an abrupt temperature, the satisfactory temperature range is bracketed. Although our terrestrial experience is a 100 °C temperature range before boiling commences for our atmospheric pressure (at sea level), this will not be the case on other planets unless they have surface pressures of 1 bar (10^5^ Pa). For example, on contemporaneous Mars, the typical pressure of 6 mbar allows water to boil at temperatures just slightly above freezing. At its deepest topographical location, where pressures might annually reach 10 mbar, the b.p. would still be only +7 °C., greatly constraining the limits of natural environments which are habitable. Once boiling begins, the lifetime of liquid H_2_O in that particular environment is severely diminished. 

On early earth, an atmospheric pressure of just 0.1 bar would put the b.p. at +46 °C. Higher pressures, such as 5 bar, would raise the b.p. to +150 °C, which could enable more effective hydrothermal extraction of nutrients from rocks and soils, especially if this atmosphere provided a major greenhouse effect that would produce strong heating even from an early faint sun. 

Another benefit of a significant atmosphere is if it also contains important nutrients, such as gaseous forms of the CHNOPS elements. An atmosphere can provide a semi-infinite source of its major constituents, which can enable high concentrations of available nutrients (and redox pairs) up to their saturation level in the pond’s aqueous medium (depending on the partial pressure of that gas) to be assimilated quickly. In contrast, extraction of nutrients from rock and soils can comparatively be quite slow, unless hydrothermal temperatures or acidic conditions speed the process. Discarding of waste products, in vapor form, is aided by a low atmospheric pressure and/or low partial pressure of the gas to be discarded. 

Exoplanets would have similar constraints. The scenarios for solar systems around other classes of stars will still have many of the same boundary conditions: an atmosphere favorable to the existence of a suitable solvent, such as liquid H_2_O, plus a source of nutrient elements and energy sources compatible with metabolic activities. The range of possibilities is just now beginning to be revealed, with extensive new observations of exoplanets, stellar properties, and modeling of habitability zones [79,151,152,153,154,155,156].

## 6. Conclusions

### 6.1. Summary

Although the molecular activities underpinning life are conducted at scales below detailed microscopic observation (sub-nm), and the organelles which characterize cellular life are themselves microscopic (sub-μm), there are multiple reasons to believe that the original milieu in which life originated was macroscopic in size. Hence the term, macrobiont. 

Abiotic chemical evolution and pre-cellular evolution via coded molecular replication leading to the development of cells may only have succeeded in a locale where a variety of environments were semi-isolated yet interconnected. Temporal perturbations can occasionally reduce the isolation barriers to enable reactions favorable to the rise and further evolution of living entities.

Various favorable mini-environments are distinguishable at meter scale, where gradients in chemical concentrations, temperature, gravity, solar flux and other influences can sustain the desired semi-isolation. Sufficient gradients cannot be sustained at the microscale. 

Numerous locales for macrobionts have been proposed. The macrobiont must provide a variety of functions and mini-settings. We have included a systems analysis approach to explore the function-space for favorable attributes of the small pond version of a macrobiont. Small ponds provide a wide variety of semi-isolated spatial environments which can be dynamically modified and/or episodically mixed. Trade-offs in pond size between frequency of occurrence, availability of concentrated ingredients, and lifetime have been examined, with the conclusion that there is a range of intermediate pond sizes which provide the maximum likelihood for the OoL. A potentially special region for evolutionary processes is the pond foreshore, where evaporites, organic precipitates, UV, wet/dry cycling, and mudcrack networks can come together to form semi-isolated compartments in a dynamic environment to promote progression of prebiotic syntheses. Such an environment also provides a selection pressure for organisms which can survive outside the macrobiont. 

In comparison with several other proposed locales for the OoL, the subaerial pond has several advantages and some disadvantages. Not to be excluded, however, is the possibility that more than one macrobiont scenario could occur in the universe, depending on stochastic variability and/or the nature of the planet where the spark of life occurs.

Experience gained from broad-based systems analysis techniques can be applied to the system needed to start life from inanimate matter. Abiotic chemical evolution can lead to a system in which quasi-independent subsystems are activated and synergistically operate together in ways which can promote accelerated evolution from molecular replicators to protocell and to a macrobiont-specialist cell (the _MB_C) that reproduces and evolves efficiently. Conventional biological evolution, promoted perhaps by the selection pressure of the pond’s dynamic foreshore or connectivity with an aquifer, can result in the creation of a variant, the colonizer cell (cC), whose metabolic and survival capabilities tolerate the more common environmental niches in the external world outside the MB.

Like all systems, useful lifetimes of macrobionts are limited, either when a critical subsystem becomes ineffective or when multiple subsystems are sufficiently degraded that the primary functionality of the overall system is compromised. Potential macrobionts which are ubiquitous in the planetary environment increase the probability that one or more will achieve the penultimate population of cell types and hence their spin-off of one or more colonizer cells which can create a biosphere that can take advantage of suitable environmental niches which are even more widespread on a global scale. Once a mini-biosphere is established, further adaptive radiation of metabolic, motility, and behavioral repertoires will allow invasion of yet other now-suitable environments.

### 6.2. Future Research

#### 6.2.1. Laboratory and Field Experiments

Systems analysis is not just for analyzing hypothetical past favorable conditions. It also can guide research. Although much research targets narrow niches which are important but so specialized that only a few groups are equipped to study them, there is also need for more general investigations leading to collections of data which allow assessment of which factors may lead to a successful OoL system, i.e., a macrobiont. These knowledge gaps currently hinder insight into which environments may have been adequate and others insufficient. 

As a composite of diverse fields of study, research into scenarios for the OoL has progressed greatly in less than one century, in spite of relatively nonconcerted and minimally funded effort. Early speculations have given way to substantial laboratory breakthroughs. Efforts to probe the history of the surface of planet Earth, and now Mars, have revealed much about the early geological, atmospheric, and biological history of these more accessible planets that can help develop more comprehensive investigations and analyses for the future [151]. Nevertheless, in all areas, much remains to be investigated and we currently are burdened with many remaining mysteries and uncertainties. Through a combination of field work, laboratory experiments, and modelling, future progress can be accelerated. 

Field work can be extraordinarily instructive, but also extraordinarily susceptible to alternative interpretations. Nevertheless, one of the most important investigations for both geological and geochemical processes remains what nature has provided us. Future emphasis on potential-macrobiont-like environments could be especially revealing.

Relevant laboratory experiments can be very difficult, because of impracticality of reproducing scales of space and time, as well as challenges in controlling and simulating conditions efficiently, safely, and in sufficient fidelity to be realistic. The need for experienced technical support personnel and the challenges, imperfections, and unpredictability with experimental setups, funding sustainability, test options, and so forth, all create impediments and can generate a disinclination to proceed. 

Although laboratory experiments using flow reactors is certainly a step in the right direction [90], the macrobiont can offer a far more complex interplay than can be realized by a combination of the idealized continuous stirred-tank reactors and the plug-flow reactors of the chemical engineers.

As an example of future research opportunities, it is possible to artificially produce mudcrack and mud peel topography, and a simple segment of a foreshore area could be constructed without simulating an entire pond, yet allowing testing of wet–dry and UV conditions in realistic heterogeneous terrain. Since the T- and Y-shaped crack patterns have significant interconnections, but other cracks are isolated, there is ample opportunity for differential chemical evolution as well as sporadic mixing. Using the technique of additive manufacturing (3D printing), artificial networks of mudcrack topography could be manufactured and used as testbeds for simulating multi-pot-like versions of sequential abiotic chemical evolution. Very fortunately, the scale of mudcrack polygons and the shoreline width and mudcrack span is not dependent on pond size, except for wave action, so that laboratory-based meter-scale experiments could suffice to realistically simulate what is possible in a variety of pond, lake, or tidal basin settings. 

Outdoor facilities for tests could take advantage of ponds that already exist but are no longer used for other functions, such as wastewater treatment (facultative lagoons, polishing ponds), solar energy ponds, farm ponds, quarry ponds, fishponds, etc. Such approaches would, however, necessitate careful control of any toxic substances that were being used or created, and may require the addition of herbicides and other treatments to prevent the confounding influences of biological activity. These precautions could be important even if the primary purpose were only to study inorganic chemical and physical processes, such as leaching, transport, etc. This would allow for study of pond processes themselves, but not likely for prebiotic chemistry. 

Another area of laboratory research is to parametrically characterize those prebiotic pathways that are most promising, in terms of the range of plausible ionic catalysts (divalent cations; transition element ions; etc.) including not only which ions are promotional and which are inhibitory. For example, which ions, such as Cr, Mn, Fe, Co, V, Ni, Zn, etc., as well as anions, may enhance or inhibit Cu catalytic activity if present at similar, plausible relative concentrations? 

The effect of various energies of UV radiation on reaction pathways and yields has often been studied using simple laboratory mercury lamp sources. However, the action spectra of the UV wavelengths are quite varied and indicate the need for careful spectrographically controlled irradiations [157] coupled with data on absorption by plausible environmental constituents.

Although P and N are available in planetary environments in ample supply, they are in mineral or gaseous forms that are unusable. Much has been written and studied about how they may be transformed into oxidized and reduced forms that are soluble in aqueous media and hence chemically available. In addition, much has been contemplated about how the all-essential carbon atoms may be obtained. Any geologic site that does not have accessible sources of C, P, and N atoms in useable chemical forms at reasonable concentrations cannot be a potential-macrobiont for LAWKI.

Computational models can be extremely useful in understanding known phenomena and in exploring the range of possible phenomena, but only when underpinned by theoretical thoroughness and verification by empirical observations and/or experimental results. Because modeling is cleaner, faster, and easily subject to refinement, it is an attractive area of research. However, it cannot and should not be used to eliminate verification in laboratory experiments of credible candidate scenarios advocated for the OoL.

Combining two or all three approaches is one of the most productive and accepted methodologies of systems analysis. For example, in the case of a pMB, once many germane interactions between element and ion concentrations are characterized under varying physicochemical conditions, and sufficiently understood, calculational exploration of the n-spaces of factors (pH, Eh, I, etc.) can be explored to place constraints on potential MB environments.

#### 6.2.2. Macrobionts as Biosignatures

An attempt could be made to search for macrobionts as biosignatures. Microscopic verification of taphonomic evidence of microbial cellular life is notoriously difficult. For this reason, it may be challenging to establish the stage of success if a fossilized macrobiont is somewhere discovered. It is however worth speculating that if a potential macrobiont were found and if the geochemical record indicated favorable conditions, especially with the presence of organic compounds indicating some degree of prebiotic evolution, the case for the origin of life in such an environment would be bolstered. Although the likelihood of such preservation in view of all the forces of obliteration on planet Earth is exceedingly small, it may be more likely found on a less active planet such as Mars.

Today, what might Darwin say? 

Perhaps, “But if (and oh what a big if) we could find some dried up little basin on Mars with all sorts of nitrate and phosphoric salts with protein residues and other biosignatures.”

## Figures and Tables

**Table 1:** Holistic viewpoints in Systems Analysis perspectives.

Perspective	Definition	Applied to Macrobiont
Context	What are the broader circumstances?	Environmental Factors
Operational	What does the system accomplish?	Origin of Life; Seeding the Biosphere
Functional	How does it accomplish these functions?	Prebiotic and biological evolution
Structural	How it is constructed and organized?	Semi-isolated compartmentalization
Generic	In what class is it of similar systems?	Inanimate geologic settings
Continuum	Is this just one of many alternatives?	Multiple proposed scenarios
Temporal	What is its past, present, and future?	Enabling extended evolution
Quantitative	What numeric measures/quantifications?	Physics, chemistry models
Hypotheses	What generalizations can be envisioned?	Exoplanetary bodies

**Table 2:** Contributors to the macrobiont environment.

Source	Chemical	Physical
Geosphere	Fundamental elements (C, P, S, NOx) Other: Mg, Ca, K, Fe, Mn, Zn (V, Co, Ni, Cu, Mo) Key minerals (clays, carbonates, volcanic ash)	Gravity field; basins for ponds; earthquakes volcanism; geomagnetic field temperature (magmatic)
Hydrosphere	H_2_O and OH reactivity Solvated gases and ions (bioavailable) pH, Eh	Liquid: solvent; fluid; volatile; adsorb; cyclable I, k, c, η, ϕ, γ, σ, T, P, ice, vapor, sol/gel braided streams, waterfalls, whirlpools
Atmosphere	Volatiles CO_2_, CH_4_, H_2_O; HCN?; N_2_, NH_3_?, H_2_; H_2_S, SO_2_	Weather: rain, clouds, wind, abrasion, saltation lightning, vortices, microbursts Thermal modifiers (greenhouse; climate)
Astronosphere	Carbonaceous chondrites; Comets (poly HCN) Organics, N, P, S, trace elements Photochemical: Sunlight Vis, IR, UV, EIR	Temporal: diurnal, seasonal, Milankovitch cycles wet/dry, freeze/thaw, light/dark temperature; GCR/SPE; GMF

I = ionic strength, k = thermal conductivity, c = specific heat, η = viscosity, σ = conductivity (electrical), ϕ = osmotic coefficient, γ = surface tension, T = temperature, P = pressure GCR/SPE = ionizing radiation from galactic cosmic rays and solar particle events; GMF = geomagnetic field.

**Table 3:** Scaling relationships for pond shapes and factors.

Pond Attribute	Scales as	Cylinder	Cone	Spherical Cup
Contact with soil, rock	Non-planar area (soil)	(π/4) D^2^ (1 + 4α)	(π/4) D^2^ Q_1_	π α D^2^
Contact with atmosphere	Planar area (atmos.)	(π/4) D^2^	(π/4) D^2^	π α D^2^ (1 − α)
Number of Cells in Pond	Volume	(π/4) α D^3^	(π/12) α D^3^	(π/3) α^2^ D^3^ (1.5 − α)
Lifetime against evaporation	Volume/atmos. area	d = α D	d/3 = α D/3	(αD/3) (1.5 − α)/(1 − α)
Lifetime against seepage	Volume/soil area	(αD)/(1 + 4α)	α D/(3 Q_1_)	(α D/3) (1.5 − α)
Foreshore (Shoreline area)	Δ non-planar area	π D Δd	(π/4) Q_1_ Q_2_ ·D ·Δd/α	~π D Δd
Mineral concentration pond volume	Soil area/volume	(1 + 4α)/(α D)	3 Q_1_/(α D)	3/[(α D) (1.5 − α)]
Mineral conc × foreshore area	Mineral conc × Shore area	π D^2^ (α + 0.25)	~(Δd/α^2^) (Q_1_)^2^ ·Q_2_	Δd/[α (1.5 − α)]
Atmospheric input to concentration	Planar area/volume	1/(α D)	3/(α D)	[3/(α D)] (1 − α)/(1.5 − α)
Atmos input conc × foreshore area	Atmos. conc × Shore area	π Δd/α	~(Δd/α^3^) Q_1_ Q_2_	(Δd/α) (1 − α)/(1.5 − α)

Q_1_ = [1 + 4α^2^]^0.5^ --> 1 + 2 * α^2^ for small α, and --> 1 for very small α; or, = 1.414 for d = D/2. Q_2_ = [1 + 0.5 (Δd/d)] --> 1 for small Δd/d, which typical (e.g., 1 cm rain into ≥10 cm deep pond).

**Table 4:** Factors of merit favoring pond size (● is major factor, O is minor).

	Parameter	Smaller	Larger
A	Number of Ponds (pMBs)	●	
B	Replicator Population		●
C	Semi-Isolated regions		●
D	Global Foreshore area	O	O
E	Pond Longevity		●
F	Nutrient concentrations	●	
F	Redox Couples (for Energy)	●	
G	Concentrations Foreshore	●	O
H	Meteorites into ponds	●	O
I	Pond peak temperature	●	
J	EUV attenuation		●
K	Meteorological stirring	●	

## Glossary

ATLO	Assembly Test and Launch Operations
cC	colonizer cell
CHNOPS	chemical abbreviations of the 6 elements essential for LAWKI
EDAC	Error detection and correction
Eh	oxidation potential
FOM	figure of merit (Section 3.5)
HTV	hydrothermal vent
ISFD	integral size frequency distribution (pond diameters)
LAWKI	Life as we know it
LUCA	Last Universal Common Ancestor
MB	Macrobiont
_MB_C	Macrobiont Cell
OoL	Origin of Life
pMB	potential Macrobiont
V.P.	vapor pressure
